# Mechanism of complement inhibition by a mosquito protein revealed through cryo-EM

**DOI:** 10.1038/s42003-024-06351-x

**Published:** 2024-05-27

**Authors:** John F. Andersen, Haotian Lei, Ethan C. Strayer, Van Pham, José M. C. Ribeiro

**Affiliations:** 1 NIH-NIAID, Laboratory of Malaria and Vector Research, Rockville, MD USA; 2 NIH-NIAID, Research Technologies Branch, Bethesda, MD USA; 3 Biological and Biomedical Sciences Program, Yale University, New Haven, CT USA

**Keywords:** Cryoelectron microscopy, Complement cascade

## Abstract

Salivary complement inhibitors occur in many of the blood feeding arthropod species responsible for transmission of pathogens. During feeding, these inhibitors prevent the production of proinflammatory anaphylatoxins, which may interfere with feeding, and limit formation of the membrane attack complex which could damage arthropod gut tissues. Salivary inhibitors are, in many cases, novel proteins which may be pharmaceutically useful or display unusual mechanisms that could be exploited pharmaceutically. Albicin is a potent inhibitor of the alternative pathway of complement from the saliva of the malaria transmitting mosquito, *Anopheles albimanus*. Here we describe the cryo-EM structure of albicin bound to C3bBb, the alternative C3 convertase, a proteolytic complex that is responsible for cleavage of C3 and amplification of the complement response. Albicin is shown to induce dimerization of C3bBb, in a manner similar to the bacterial inhibitor SCIN, to form an inactive complex unable to bind the substrate C3. Size exclusion chromatography and structures determined after 30 minutes of incubation of C3b, factor B (FB), factor D (FD) and albicin indicate that FBb dissociates from the inhibited dimeric complex leaving a C3b-albicin dimeric complex which apparently decays more slowly.

## Abstract (web summary)

Structural analysis using cryo-EM reveals that albicin, an inhibitor of the alternative pathway of complement from the saliva of a mosquito, binds the C3bBb complex through contacts with both chains of C3b as well as the protease factor B.

## Introduction

Complement is an arm of the innate immune system whose activation results in opsonization of pathogens prior to phagocytosis and direct killing through formation of lytic membrane attack complexes^1^. Additionally, the proinflammatory, vasoactive anaphylatoxins C3a and C5a are byproducts of proteolytic cleavage of complement factors C3 and C5. Three distinct pathways of complement activation, the classical (CP), lectin (LP) and alternative (AP), are initiated by different sets of effector molecules but converge at the point of the proteolytic C3 convertase (C3bBb) of the alternative pathway which cleaves the scaffold protein C3 to form C3b.and anaphylatoxin C3a^1^. The convertase is formed when the serine protease zymogen factor B (FB) binds with C3b that is covalently attached to pathogen or tissue surfaces. The link is formed through reaction of an unstable thioester structure in C3b with surface nucleophiles. C3b-bound FB is cleaved by a second serine protease, factor D (FD), to give its active form, FBb. Despite loss of the FBa domain of FB, the FBb fragment containing the von Willebrand A (vWA) and serine protease (SP) domains remains bound to C3b through ligation of the C-terminal carboxylate of C3b to a magnesium ion at the metal ion-dependent adhesion site (MIDAS) in the vWA domain^2,3^. Uncontrolled complement activation is responsible for a variety of human diseases including atypical hemolytic uremic syndrome (aHUS), paroxysmal nocturnal hemoglobinuria (PNH) and age-related macular degeneration. Inhibitors such as the monoclonal antibody product eculizumab have been effective in treating these disorders and the development of biologic and small molecule complement inhibitors remains an active area of investigation^4,5^.

Inhibition of the host complement system is also a key factor for successful blood intake by mosquitoes, sand flies, ticks and other blood feeding arthropods^6–10^. Activation of opsonins and the membrane attack complex (MAC) can cause damage to tissues of the digestive system of a blood feeder which may not protected by the complement control systems of host tissues. Additionally, anaphylatoxins produced during activation cause vascular leakage and rapid activation of skin mast cells and leukocytes which may make feeding more difficult. Complement activation has also been shown to be important for antibody mediated killing of *Plasmodium* parasite stages within the mosquito making it a consideration in the development of transmission blocking vaccines^11^.

Naturally occurring protein inhibitors of complement have been identified in a diverse group of blood feeding arthropods that serve as examples of potentially novel inhibitory mechanisms^6–10,12^. Proteins and peptides from different species have been identified which interact with the CP, AP, LP and terminal pathways of complement. Tick saliva has been a rich source of complement inhibitory proteins and peptides. Many of these (RaCI, CirpT1 and OmCI) target C5, blocking its activation and preventing formation of the MAC and production of anaphylatoxin C5a^9,12,13^. Other inhibitors (Isac, Irac I and CirpA) target the AP by scavenging properdin, a protein that stabilizes the C3 proconvertase and convertase^10,14,15^. Scabies mites have been shown to produce protease-like molecules that inhibit the CP and LP directly by binding C1q and mannose binding lectin^16^.

In blood feeding Diptera, proteins targeting the CP and AP have been identified. SALO, a protein from saliva of the sand fly *Lutzomyia longipalpis* has been shown to block the CP at an early point in the pathway that has not been identified^6^. More recently, inhibitors from sand flies and mosquitoes have been shown to inhibit the AP by forming stable complexes with assembled C3 convertase components. Lufaxin from the sand fly, *L. longipalpis*, binds the C3 proconvertase complex and stabilizes the interaction between C3b and FB while at the same time preventing a conformational change that is essential for conversion of C3bB to C3bBb^17,18^. Proteins from the mosquito Sg7 protein family, albicin from *An. albimanus* and Sg7AF from *An. freeborni*, act by blocking the cleavage of C3 by the C3 convertase, C3bBb. Salivary gland extract from *An. aquasalis* also inhibited the AP and and a homologous component from this species is considered likely to act by the same mechanism^7,8,19^. Albicin and its orthologs promote formation of a complex containing C3b and FBb on properdin coated surface plasmon resonance surfaces. Size exclusion chromatography revealed a considerable increase in the size of the inhibited complex relative to C3bBb, suggesting oligomerization of the convertase^7,19^. The analysis has also verified that the large complex contains albicin as a bound component. These characteristics suggest that these mosquito inhibitors act similarly to the bacterial inhibitor SCIN by inducing formation of C3 convertase dimers^20^.

In this study we examine the mechanism of complement inhibition by albicin. Using single particle cryo-EM we have determined structures of the AP C3 convertase containing bound albicin. Structures determined from a single sample vitrified approximately 30 min after addition of FD, along with gel filtration chromatography studies, suggest that in solution an inhibited complex containing dimeric albicin-C3bBb forms initially, followed by dissociation of FBb to form a dimeric albicin-C3b complex which then dissociates to form its individual components. The complex is structurally quite similar to the SCIN-bound C3bBb dimer but the binding modes of the inhibitors differ significantly and the inhibitor proteins are not evolutionarily related as indicated by a lack of sequence conservation^21–23^.

## Results

### Formation and stability of the C3bBb-albicin complex

**Fig. 1:**
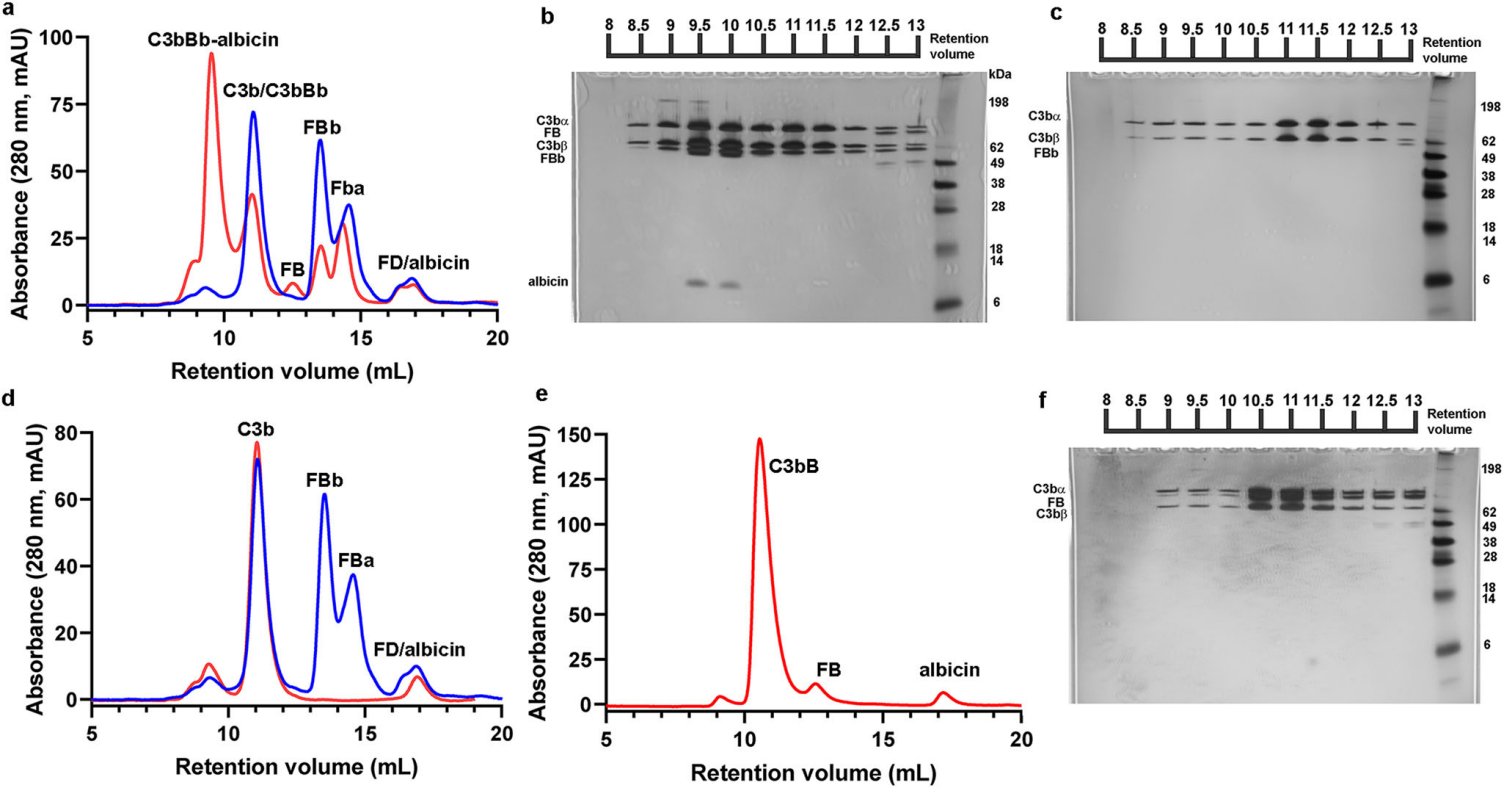
Formation of albicin inhibited C3bBb. Size exclusion chromatography on Superdex 200 of reaction products after incubation of C3b, FB and albicin with FD. **a** Comparison of complex formation after 1 h (red) and 14 h (blue). **b** SDS-PAGE of fractions from panel a, red trace. The dimeric peak (C3bBb-albicin) contains all components of the complex. **c** SDS-PAGE of fractions from panel **a**, blue trace. Highest molecular weight peak contains only C3b. **d** Size exclusion traces showing a C3b-albicin mixture (red) and fully dissociated C3bBb-albicin (blue). Albicin does not induce significant dimerization of C3b. **e** Formation of C3bB in the presence of Ni^2+^ and albicin. **f** SDS-PAGE of fractions from panel **e**. C3b and FB but not albicin are seen in the peak labeled C3bB.

We reconstituted the inhibited C3bBb complex in solution by incubating C3b, FB and albicin with FD at room temperature in the presence of magnesium. Size exclusion chromatography of the mixture showed formation of a high molecular weight complex after 1 h which dissociated completely by 14 h (Fig. 1a–c). SDS-PAGE of fractions collected after 1 h incubation showed that albicin was associated with the high molecular weight component that also contained both chains of C3b and FBb (Fig. 1a, b). The high molecular weight peak shows a smaller retention volume than C3bBb formed in the absence of albicin^19^ and apparently represents an oligomeric form of the C3bBb complex. When albicin was incubated with C3b for 1 h, no oligomeric complex or bound albicin was associated with the C3b containing fractions (Fig. 1d, Supplementary Fig. S1). The apparent low affinity of albicin for monomeric C3b was verified using surface plasmon resonance (SPR) which showed an affinity 0.8 µM for the binding of albicin with an immobilized C3b surface (Supplementary Fig. S1d, e). We also formed the proconvertase, C3bB, in the presence of albicin and Ni^2+^ (which stabilizes C3bB) and found that no albicin was present in size exclusion fractions containing the protein complex, indicating that albicin does not block the binding of FB with C3b or directly associate with C3bB itself (Fig. 1e, f). Overall, these experiments confirm that albicin binds C3bBb and stabilizes it, but its affinity for the C3b component alone is much lower. High affinity binding only occurs in the presence of FBb and formation of the C3bBb-albicin complex induces oligomerization. To further understand the structural basis for these observations we formed the C3bBb-albicin complex in solution by incubating C3b, FB and albicin for 30 min with FD in the presence of magnesium and analyzed its structure by cryo-EM.

### Particle classes and overall structure of the C3b-albicin complex

**Fig. 2:**
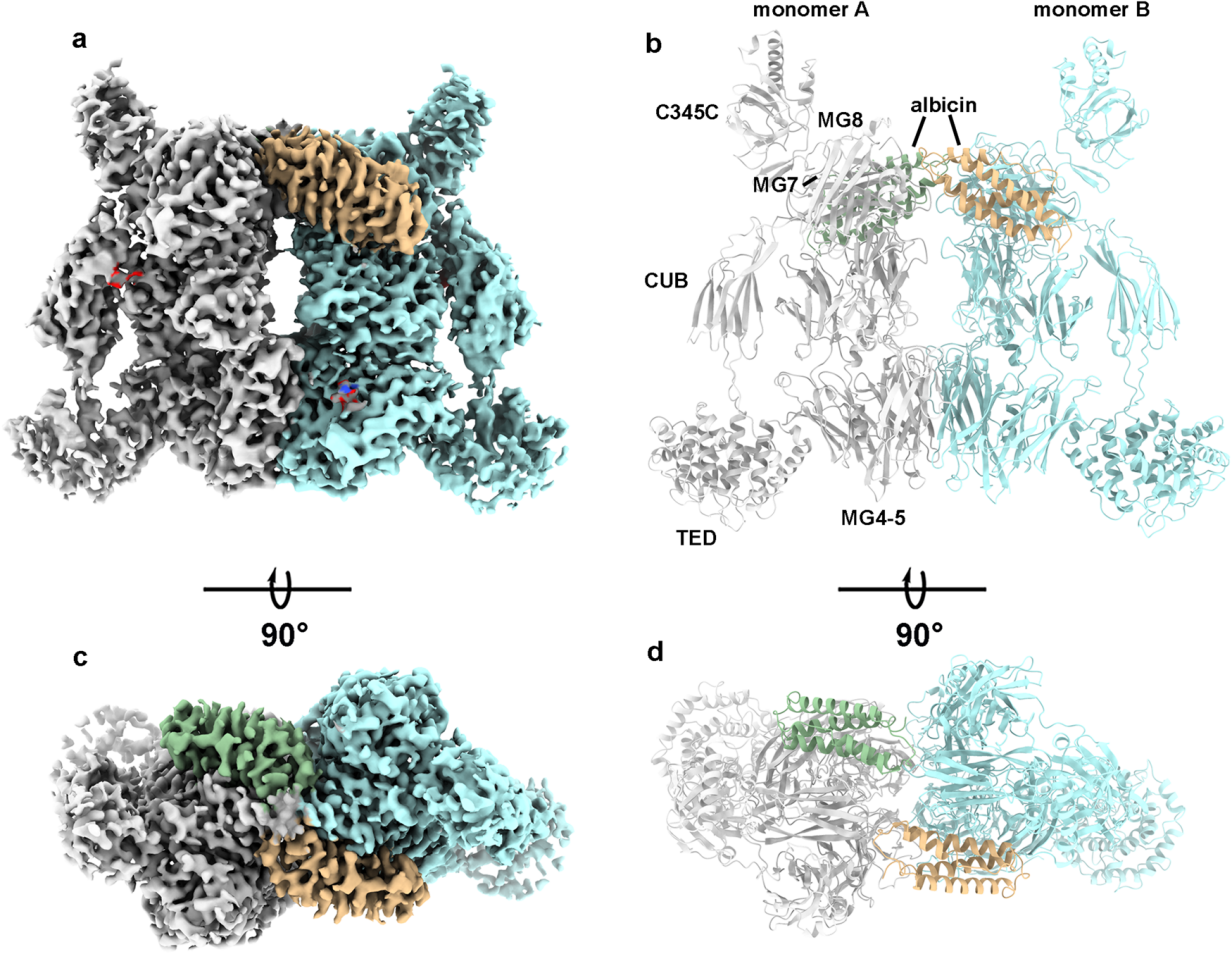
Cryo EM map and with a fitted model of C3b-albicin obtained after 30 min incubation of reaction mixture prior to vitrification. **a**, **c** Two views of the refined map representing the C3b-albicin dimeric structure. Density corresponding to the C3b monomers is colored in gray and blue. Density corresponding to bound albicin is colored in green and wheat. **b**, **d** Ribbon diagrams of C3b-albicin in the views shown in panels **a** and **c**. Domain structures are labeled in monomer A.

After template-guided particle picking, 3D classification yielded four classes containing dimeric C3b structures resembling those seen in crystal structures of the C3bBb complex inhibited with the bacterial protein SCIN^22^ (Supplementary Fig. S2, Table 1). In all four classes, the C3b monomers are related by two-fold symmetry around an axis running parallel to the broad surface of the particle (Fig. 2, Supplementary Fig. S2). The classes differ in containing either no FBb (class 4 in Supplementary Fig. S2), one bound FBb molecule (classes 1 and 2 in Supplementary Fig. S2) or two bound FBb molecules (class 3 in Supplementary Fig. S2). Class 2 exhibited a low-resolution reconstruction and was not analyzed further, while the class 1 particle set produced a reconstruction containing one bound FBb molecule and was refined to a resolution of 4.42 Å. Class 3 and class 4 produced the highest resolution reconstructions which were fully refined as representatives of the C3bBb-albicin dimer (class 3 in Supplementary Fig. S2) and the C3b-albicin dimer (class 4 in Supplementary Fig. S2) and will be described below.

**Table 1:** Cryo-EM data collection and model fitting for the C3b-albicin dimer and C3bBb-albicin dimeric structures

	C3b-albicin dimer PDB-8UH2, EMD-42244	C3bBb-albicin dimer PDB-8UIN, EMD-42300
Data collection		
Magnification	45,000	45,000
Voltage (kV)	200	200
Total electron exposure/used (e/Å^2^)	66.73	66.73
Defocus range (µm)	−0.3 to −2.2	−0.3 to −2.2
Pixel size (Å)	0.44	0.44
Symmetry imposed	C2	C2
Initial particle images	492,352	492,532
Final particle images	105,782	65,862
Map Resolution (Å)	3.59	3.86
(FSC threshold)	0.143	0.143
Refinement		
Initial model used	6RUR, 6XKE	6RUR, 6XKE
Map sharpening *B* factor (Å^2^)	−152.5	−203.5
Model composition		
Non hydrogen atoms	24,894	28,249
Protein residues	3348	3746
Ligands	4	5
*B* factors (Å^2^)		
Protein	158.47	127.34
Ligand	90.86	134.17
r.m.s. deviations		
Bond lengths (Å)	0.006	0.003
Bond angles (°)	0.611	0.539
Validation		
MolProbity score	1.79	1.73
Clash score	8.9	8.9
Ramachandran plot		
Favored (%)	95.5	96.2
Allowed (%)	4.4	3.7
Outliers (%)	0.1	0.1

The dimeric C3b-albicin structure (class 4) was refined with C2-symmetry enforced, producing the highest resolution reconstruction of any of the 3D classes (Supplementary Fig. S2). The core part of the structure containing the macroglobulin domains MG1-MG7 of C3b is well ordered, while the complement C1r-C1s, UEGF, BMP1 (CUB) domains are covered by weaker density and the peripheral thioester (TED) and C345C domains are more poorly ordered due to their high flexibility (Fig. 2). The symmetrical dimer interface is formed by interaction of the MG4 and MG5 domains from each C3b monomer (referred to as monomers A and B) as is seen in structures of C3bBb containing SCIN (Fig. 2a, b)^21–23^. Inhibitors such as compstatin and CRIg also bind at this surface of C3b and prevent its interaction with the C3 substrate^1,24,25^. It has been proposed that that MG4 and MG5 form an exosite on C3b for binding of the substrates C3 and C5 through interactions resembling the C3b-C3b interactions seen in the structures presented here and in previously published structures of SCIN complexes^21,22,26,27^. In our map, density accommodating two molecules of albicin per dimer is located at symmetry-related positions adjacent to the C345C domains of C3b (Fig. 2c, d). The interaction surface of each albicin molecule contacts both molecules of C3b in the dimer. Although generally similar in its binding site to that of SCIN, the contact points of albicin with C3b differ from those of the bacterial inhibitor due to surface differences in the two types of inhibitors. This result shows that the C3b-albicin dimer is sufficiently stable to be detected in the absence of FBb even though FBb binding strongly favors dimer formation as shown by size exclusion chromatography (Fig. 1a, b, d). Apparently, FBb dissociates leaving the albicin-C3b dimer which then dissociates more slowly as indicated by size exclusion chromatography measurements after 14 h of incubation (Fig. 1a, c).

### The albicin-C3b binding interface

**Fig. 3:**
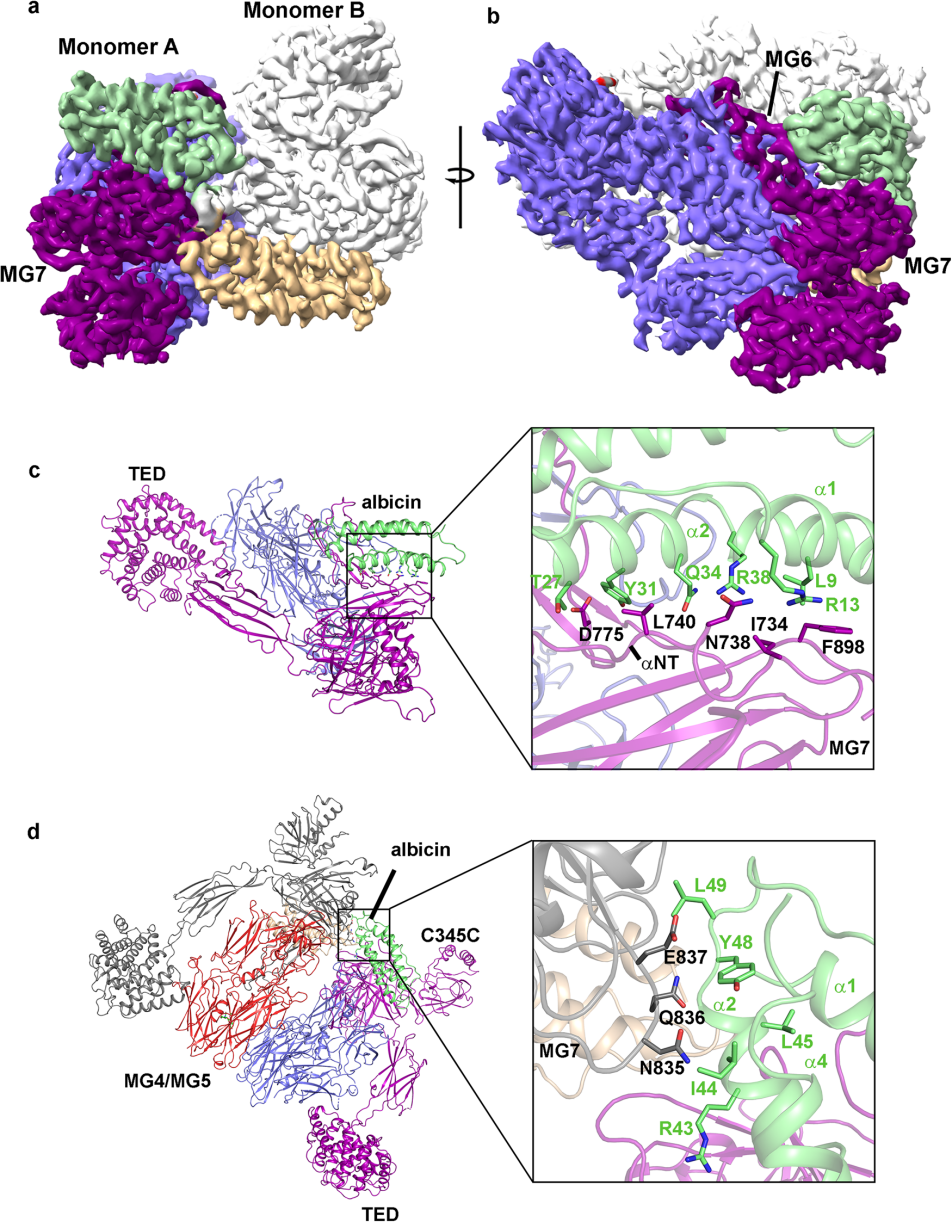
Detail of the dimeric C3b-albicin binding interface. **a** Cryo-EM map obtained after localized refinement. Density corresponding to two symmetrically arranged albicin molecules (green and wheat) contact sites on the α-chain (purple in monomer A) and β-chains (blue) of C3b. Each inhibitor also contacts a site on the second monomer. **b** Map in panel A rotated ~80° around the axis shown. **c** Ribbon diagram showing details of the α1-α2 face of albicin (green) contacting the MG7 domain and αNT (purple) of monomer A. Side chains observable in the map are shown in the expanded figure as sticks with oxygen colored red and nitrogen blue. **d** Ribbon diagram of dimeric C3bB-albicin highlighting the interface of albicin (green) with MG7 of monomer B (gray). Side chains are shown in the expanded figure as in panel **c**.

After particle subtraction using a mask corresponding to the TED, CUB and C345C domains of the dimeric C3b-albicin complex, the remaining core structure containing the macroglobulin domains of C3b and albicin from the C3b-albicin dimer structure (class 4) was refined using localized refinement with C2 symmetry enforced giving a map with local resolution of 3.1–3.5 Å at the C3b-albicin interface (Fig. 3, Supplementary Fig. S3). The interface includes solvent excluded surface areas of 1153 Å^2^ for albicin and 1188 Å^2^ for C3b (Supplementary Table S1). The C3b binding region of albicin is comprised of portions of α-helices α1, α2 and α4 as well as the α2-α3 loop (Fig. 3a–d). The inhibitor sits in a groove between MG6 and MG7 of C3b (monomer A) which also includes the N-terminal part of the α-chain (αNT, Fig. 3a–c). The α1-α2 face of albicin crosses over the αNT segment of C3b and interacts with the C-terminal β-strand of MG7 (Fig. 3a–c). The side chains of Tyr 31, Gln 34 and Arg 38 from α2 contact C3b residues in the region of Asn 738-Arg 740 in the αNT segment of C3b (Fig. 3c, Supplementary Fig. S4a). Several α2 residues having hydrophobic side chains, including Leu 9, also interact with MG7 around the side chain of Phe 898 (α-chain, Fig. 3c). The α2-α4 face of albicin crosses over MG6 of C3b with Tyr 31 (α2) interacting with Asp 775 in the α-chain of C3b and the side chains of Thr 35 and Gln 36 (α2 of albicin) interacting with the β-chain of C3b at Gln 557 (Supplementary Fig. S5). Phe 772 of the α-chain of C3b is also in position to contact Leu 100 in α4 (Supplementary Fig. S5). Finally, the bridging interaction between albicin and monomer B of C3b is formed by interaction of the C-terminal end of α2 and the α2- α3 loop of albicin (Arg 43-Leu 49) with the MG7 region between Asn 835 and Leu 838 (Fig. 3a, d, Supplementary Fig. S4b).

### The albicin FBb interface

**Fig. 4:**
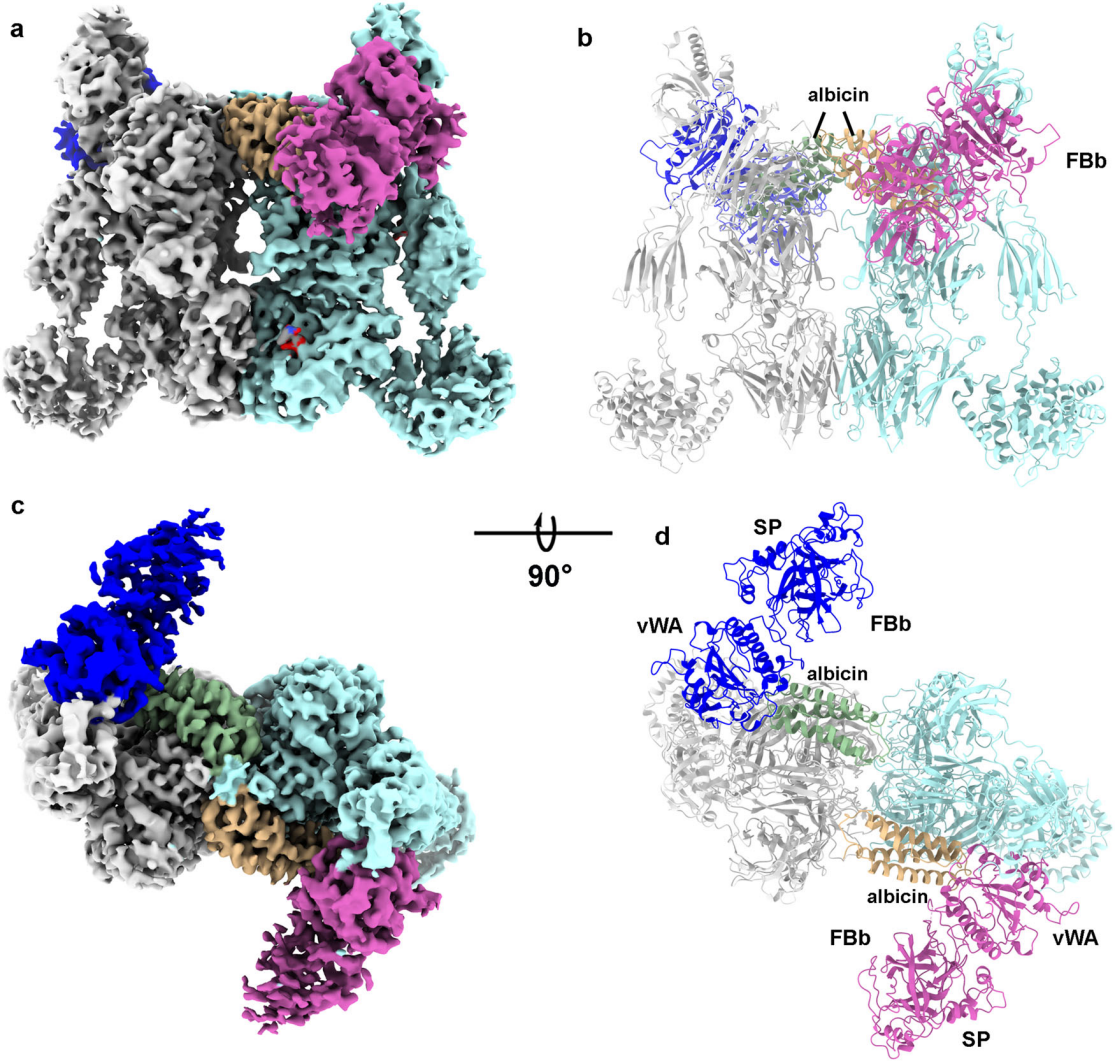
Cryo EM map of and fitted model of C3bBb-albicin obtained after 30 min incubation of reaction mixture prior to vitrification. **a**, **b** Two views of the refined map representing the C3b-albicin dimeric structure. Density corresponding to the C3b monomers is colored in gray and light blue. Density corresponding to bound albicin is colored in green and wheat. Density for FBb is colored dark blue and magenta **c**, **d** Ribbon diagrams of the C3bBb-albicin model corresponding to the views shown in panels **a** and **b**.

**Fig. 5:**
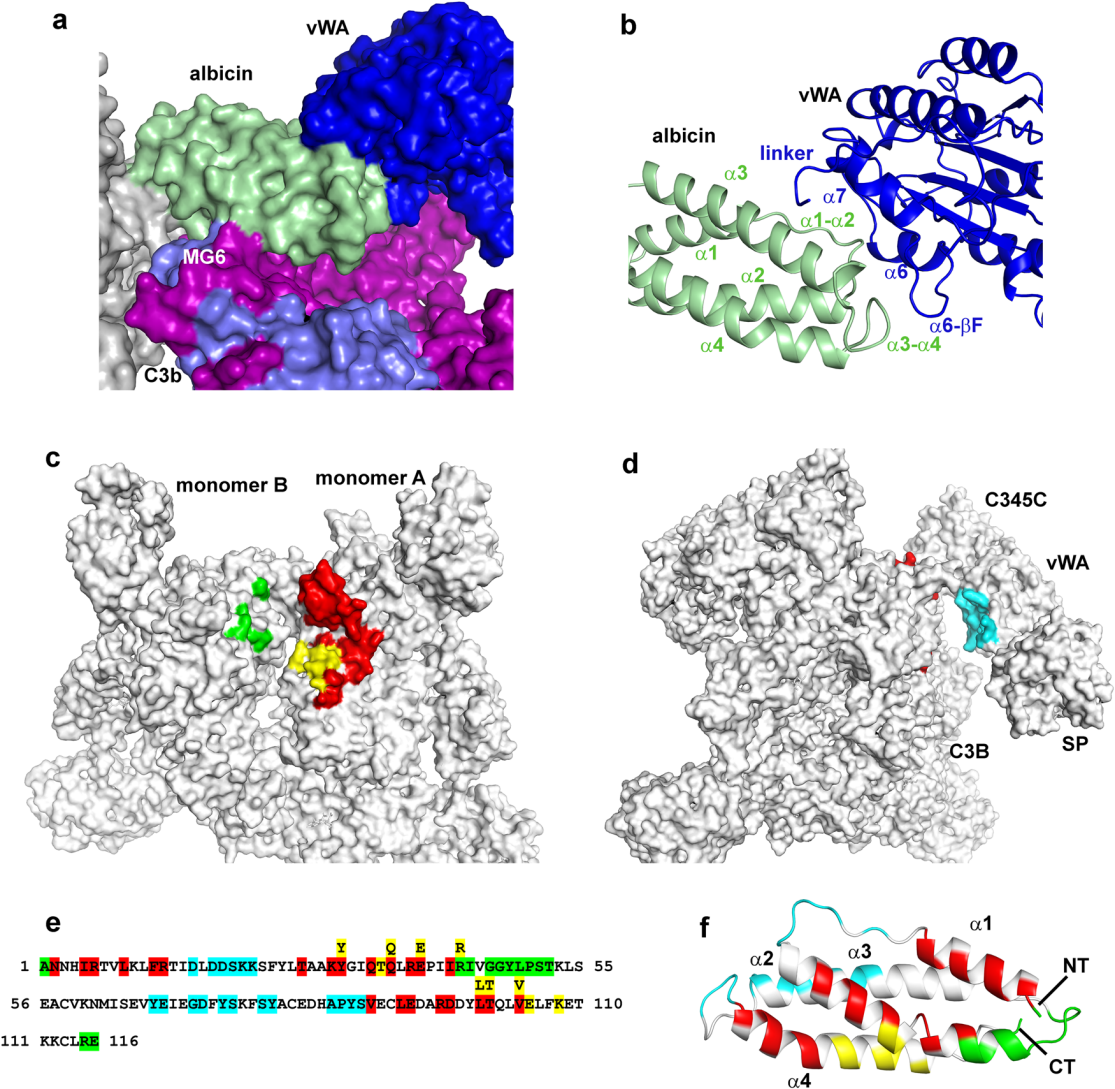
The albicin-FBb interface and a summary of albicin interactions with C3b and C3bBb. **a** Surface representation of the C3bBb-albicin dimeric model showing interaction with C3b (β-chain in blue and α-chain in purple) and FBb (vWA domain in blue). **b** Ribbon diagram showing albicin FBb (vWA) interface region colored as in panel **a**. **c** Surface representation C3b dimer model (albicin and FBb removed) to show albicin-C3b interaction surfaces colored by C3b chain (yellow, β-chain, monomer A; red, α-chain, monomer A; green, α-chain, monomer B). **d** Model surface of C3bBb dimer with albicin removed to show FBb albicin interface colored in cyan. **e** Amino acid sequence of albicin with C3bBb-interacting residues colored as in panels **c** and **d**. Residues interacting with multiple chains are shown in a second row of sequence. **f** Ribbon diagram of albicin with residues colored by interaction site on C3bBb (NT N-terminus, CT C-terminus) in the coloring of panels **c** and **d**.

In the reconstruction of 3D class 3, density covering two molecules of FBb was observed after refinement applying C1 symmetry, allowing the structure to be further refined with C2 symmetry enforced (Fig. 4a–d, Supplementary Figs. S2, S6, Table 1). The von Willebrand factor A (vWA) domain of FBb shares an extensive binding interface with albicin and its secondary structural elements are well defined along with the side chains of bulky and buried amino acids (Fig. 5a, b, Supplementary Fig. S7). Density for the SP domain is much weaker, making it difficult to visualize side chain and main chain details. The C-terminal end of the C345C domain of the C3b α-chain, which normally interacts with a magnesium ion at the vWA MIDAS, is also disordered making it difficult to verify that the MIDAS contains bound magnesium and if the C3b α-chain is associated with the metal.

We analyzed the vWA-albicin interface by masking the peripheral regions of the complex containing the TED, CUB and the C345C domains of C3b along with the SP domain of FBb, followed by particle subtraction (Supplementary Fig. S6c–f). Localized refinement was performed on the remaining volume containing the core regions of C3b, albicin and the vWA domains of FBb from monomers A and B, resulting in a map having a local resolution of 3.4–4.0 Å at the albicin-C3b interfaces and 3.9–4.5 Å at the albicin-vWA interface which includes 558 Å^2^ of buried albicin surface and 530 Å^2^ of FB surface (Fig. 5a, b, Supplementary Fig. S6c–e, Supplementary Table S1). The albicin portion of the FBb binding interface is made up of the C-terminal end of α3, loop α3-α4 and loop α1-α2 (Fig. 5a, b, Supplementary Fig. S8). The vWA portion of the interface contains, α6, the α6-βF loop, βF, α7 and the vWA-SP linker (Fig. 5b). The α1-α2 loop of albicin lies adjacent to α6 and βFof vWA while the α3-α4 loop of albicin interacts with residues at the apex of the α6- βF loop of the vWA domain and α3 interacts with α7 and the SP linker (Fig. 5b). The properdin binding site described in previous crystal structures of C3bB-SCIN in complex with an engineered two-chain properdin variant^21^ is not blocked by albicin, consistent with the enhancement of complement inhibition seen in the presence of added properdin from the albicin variant from the mosquito *An. freeborni*^19^. Taken together, analyses of the C3b-albicin and C3bBb albicin dimeric structures show an interface that includes the α and β chains of both monomers of C3b as well as the vWA domain of FBb (Fig. 5c, d). These contact the albicin structure at points that include residues distributed over the entire length of the albicin sequence (Fig. 5e, f).

### The binding mechanism of albicin

**Fig. 6:**
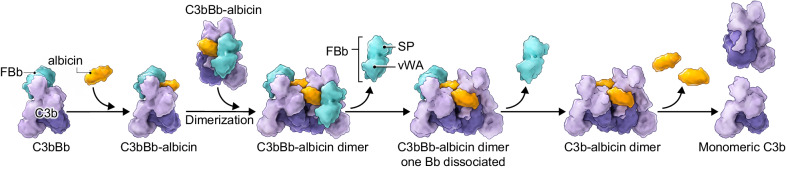
Proposed binding mechanism for albicin with C3bBb in the fluid phase. On the left albicin is shown binding with C3bBb. The complex then dimerizes by addition of a second C3bBb complex giving the C3bBb-albicin structure shown in Figs. 4 and 5. FBb then dissociates from the complex producing complex with a single FBb molecule bound as represented by classes 1 and 2 from 3D classification. Dissociation of the second FBb molecule then produces the C3b-albicin dimeric structure shown in Figs. 2 and 3. Weakly bound albicin then dissociates leading to dissociation of the dimeric C3b structure as indicated in the size exclusion chromatography experiment after 14 h incubation shown in Fig. 1a.

We have resolved three structurally distinct intermediates of albicin-bound dimeric convertase products from a single preparation. Size exclusion chromatography experiments show that in solution, albicin is associated exclusively with oligomeric complexes which cryo EM experiments indicate are C3b-albicin and C3bBb-albicin. Although SPR analysis shows relatively weak binding (K_D_ = 795 nM) of albicin to immobilized C3b (Supplementary Fig. S1), chromatographic experiments show that albicin does not remain bound to C3b during chromatography or induce formation of a stable dimer in the absence of FBb, suggesting that the observed C3b-albicin dimers are largely derived from C3bBb-albicin dimers as a result of FBb dissociation. A dissociation constant of 177 nM was reported by Ricklin et al. for SCIN binding with immobilized C3b by SPR and they also demonstrated competition FB and FH binding sites by SCIN in solution^28^. Additionally, Garcia et al. were able to obtain a dimeric C3bBb-SCIN crystal structure in the absence of FBb^26^ further suggesting that SCIN binding to C3b monomers may be significant. The presence in our cryo-EM preparations of particle classes containing two (20%), one (48%) or no (32%) bound FBb molecules, with apparently full occupancy of albicin, suggests that FBb dissociates more quickly from the complex than albicin (Supplementary Fig. S2). The multiphasic dissociation kinetics previously observed for C3bBb-albicin complexes assembled on properdin SPR surfaces are also consistent with a sequential dissociation process^19^. Since albicin has a low affinity for C3b we propose that it initially binds the monomeric C3bBb complex and induces dimerization with a second abicin-C3bBb complex through the bridging interactions between monomers A and B described above (Fig. 6). This complex then decays by dissociation of the two FBb molecules, leaving the C3b-albicin dimer (Fig. 6). It is well established that FBb does not reassociate with C3b^22^, so the C3b-albicin dimer eventually dissociates to form free C3b, FBb and albicin as shown by size exclusion chromatography (Figs. 1a, c, and 6). After depletion of FB in the presence of FD, the relatively low affinities of FBb and albicin for monomeric C3b does not allow reformation of the complex and only the individual components are observed. Albicin-C3bBb and albicin-C3b dimers are apparently both present in the high molecular weight fractions of the activation mixture that contains bound albicin (Fig. 1b).

Since albicin does not bind tightly to the C3b monomer, it would not inhibit the activation of FB by blocking formation of the monomeric C3bB proconvertase, explaining the apparently normal production of FBb in the presence of albicin after activation of human serum by rabbit erythrocytes^7,19^. However, albicin partially occludes the binding surface for the Ba fragment of FB on the surface of the C3b dimer which includes MG6, MG7 and αNT of C3b^2^. This would suggest that it could inhibit formation of C3bB proconvertase complexes originating from FB binding to albicin-C3b dimers and cause a reduction in the cleavage rate of FB in this case. Apparently, the dimer-specific effect is not large enough to significantly inhibit FB cleavage in activated serum where monomeric C3b is present.

### Comparing the binding of albicin and SCIN

**Fig. 7:**
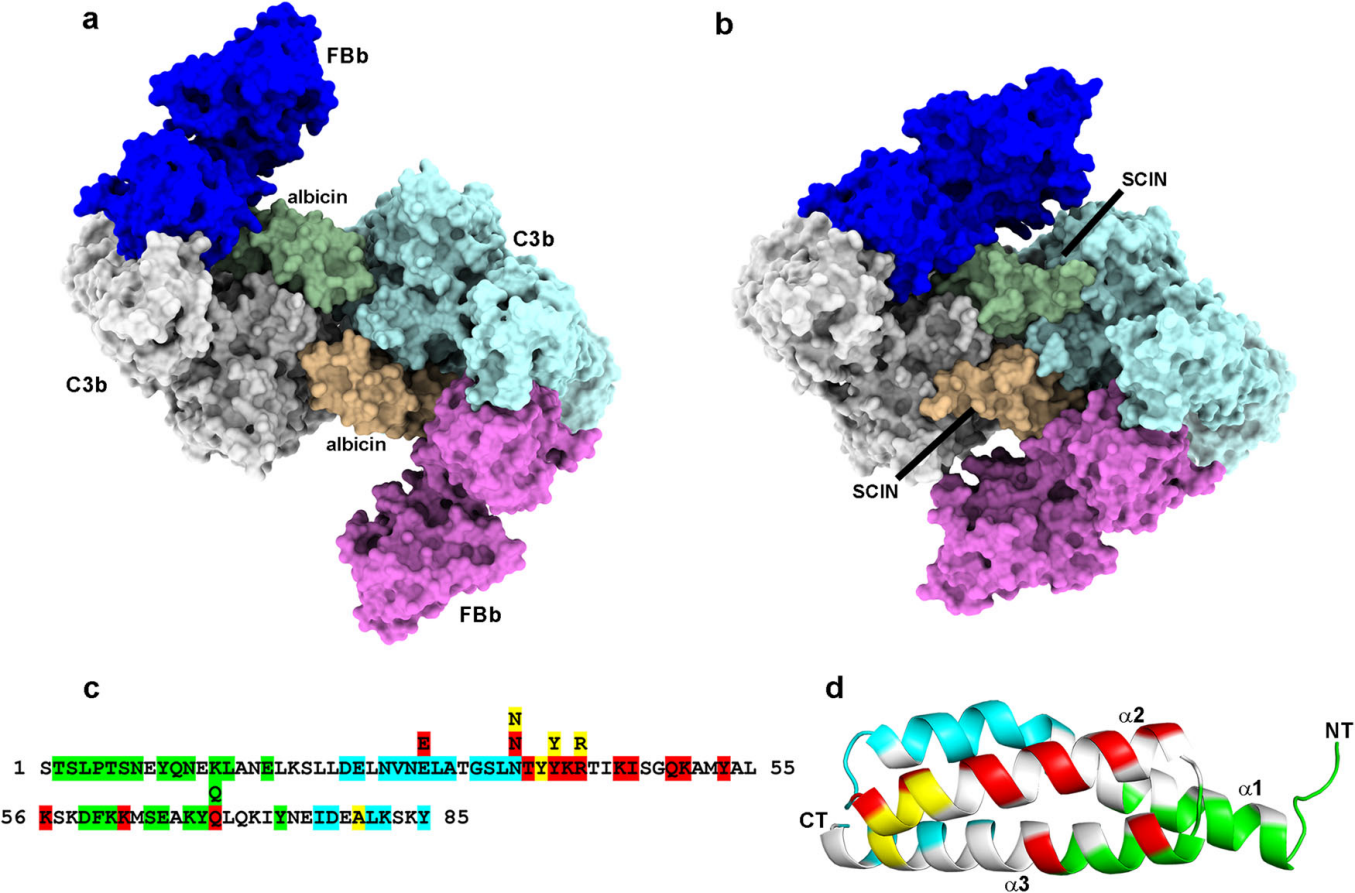
Structural comparison of albicin and SCIN. **a**, **b** Surface representations of the C3bBb-albicin (**a**) and C3bBb-SCIN (**b**, PDB accession number 2WIN^22^) models. Albicin and SCIN are colored in green and wheat, C3b monomers are colored in gray and light blue and Fb molecules are colored in dark blue and magenta. **c** Amino acid sequence of SCIN with C3bBb-interacting residues (determined using PISA and the model 2WIN) colored as in panels **c** and **d** of Fig. 5. **d** Ribbon diagram of the SCIN structure showing the C3bBb contact regions colored as in the albicin model of Fig. 5f.

Albicin and SCIN are of similar size and have superficially similar shapes but the lack of significant sequence identity (~18% in a pairwise comparison) between them indicates that anticomplement function has evolved independently in the two (Supplementary Fig S9a). SCIN has an antiparallel three-helix bundle structure with no disulfide bonds while albicin and other members of the Sg7 family are comprised of an antiparallel four-helix bundle structure with two disulfide bonds^19,20^. Both albicin and SCIN bind the C3bBb complex, interact with the C3b and FBb components and induce dimerization of C3b through bridging of the two monomers^7,22^. The binding sites of SCIN and albicin on the C3bBb surface overlap but their interactions with C3b and FBb differ significantly (Fig. 7, Supplementary Fig. S9b, c). Comparison of Figs. 5e, f and 7c, d, as well as Supplementary Table S1, show the sequence and surface distribution of residues of albicin and SCIN which contact the various chains of the C3bBb dimer as calculated using PISA^29^. The differences in the arrangements of contact points along the amino acid sequences of the two inhibitors further indicate that albicin and SCIN are not evolutionarily related and have acquired the ability to bind convertase complexes independently.

When the two complexes are compared, albicin is found to extend further along the surface of the MG6 domain of C3b than does SCIN, thereby causing FBb to tilt away from the C3b surface relative to the SCIN structure (Fig. 7a, b, Supplementary Fig. S9b, c). This feature of the structure demonstrates the flexibility of C3b-bound FBb and its ability to move in relation to C3b while still maintaining contact with the C345C domain, which is also flexible (Fig. 7a, Supplementary Fig. S9C). Like albicin, SCIN interacts with the N-terminus of the C3b α chain (αNT) but only through interfacing residues contained in α2 of the inhibitor which runs antiparallel to this portion of C3b (Supplementary Fig. S9B). In albicin, α1 and α2 both interact with the αNT segment by crossing over it. SCIN and albicin both also interact with MG6; SCIN through α2 and albicin through α2 and α4. Since albicin projects approximately 19 Å further along the C3b surface than SCIN in the direction of MG6, its interactions with MG6 are more extensive than those of SCIN (Supplementary Fig. S9b, Supplementary Table S1).

Both albicin and SCIN bind FBb in the region containing α6, βF and α7.of the vWA domain of FBb and contact the linker region between α7 of vWA and the SP domain of FBb. SCIN accomplishes this through interaction of α1 and its α1-α2 loop while albicin interacts via its α1-α2 and α3-α4 loops. In albicin, the bridging interaction between the two C3b monomers is accomplished through binding at MG7 of monomer B of C3b, while SCIN extends further over the monomer B MG7 surface by approximately 12 Å forming a much more extensive contact interface that also includes elements of MG8 (Supplementary Fig. S9b). Overall, the solvent-accessible surface buried in the albicin interface with C3b in monomer A is 1.19-fold larger than that for SCIN (1733 Å^2^ for albicin vs 1446 Å^2^ for SCIN) but the solvent accessible surface buried in the SCIN-monomer B interface is 2.8-fold larger than for albicin (1731.4 Å^2^ for SCIN vs 608.6 Å^2^ for albicin). The larger binding interface in the SCIN C3b complex relative to the albicin-C3b complex probably contributes the higher affinity of SCIN for C3b^28^. The buried surface area at the SCIN interface with FBb is also larger (1508.9 Å^2^) than the comparable interface of albicin with FBb (1088.6 Å^2^), a difference of approximately 1.4-fold.

SCIN is known to also inhibit formation of C3b by the CP C3 convertase C4b2a, in addition to inhibiting C3bBb activity while albicin does not inhibit CP activation^7^. With the information at hand, it is difficult to determine what prevents albicin from binding C4b2a but superposition of a C2a structural model (determined by X-ray diffraction, PDB ID 2I6Q) with FBb in the C3bBb-albicin model shows a glycan at Asn 447 in C2a, while FBb contains tryptophan at the corresponding position 457^3,30^. Our model indicates that the glycan would clash with albicin but not with SCIN, if the binding modes for the two inhibitors are otherwise analogous to those seen in C3bBb. Lack of glycosylation at this position is a general feature of mammalian FB as Trp 457 is conserved in human, bovine and murine forms.

### Distribution of salivary complement inhibitors in anopheline mosquitoes

The Sg7 salivary protein family is distributed throughout the genus *Anopheles* but the complement-inhibitory potency of the saliva is species dependent. Salivary gland extracts from *An. albimanus* and *An. aquasalis* have been shown to be highly inhibitory in erythrocyte lysis assays while *An. gambiae*, *An. stephensi* and *An. dirus* extracts did not show detectable activity. *An. albimanus* and *An. aquasalis* belong to the largely neotropical subgenus *Nyssorhynchus*, while *An. gambiae*, *An. stephensi* and *An. dirus* belong to the subgenus *Cellia* which occurs in Africa, the Indian subcontinent and southeast Asia. *An. freeborni* salivary gland extracts showed significant inhibition of the AP. This species belongs to the subgenus *Anopheles* which occurs in tropical, temperate and arctic regions worldwide. Recombinant albicin from *An. albimanus* and Sg7AF from *An. freeborni* target C3bBb and are potent inhibitors of the AP but Sg7AF is more effective in inhibiting lysis of rabbit erythrocytes in normal human serum containing properdin than in properdin-depleted serum^19^. Albicin, on the other hand, is equally effective in normal serum and properdin-depleted serum suggesting that it does not require the additional stabilization of C3bBb provided by properdin to form a stable high affinity inhibitory complex^7^. Recombinant *An. stephensi* Sg7-1 (anophensin^31^) also produced detectable inhibition but only at greatly elevated concentrations indicating a reduced affinity in its interaction with C3bBb^19^.

**Fig. 8:**
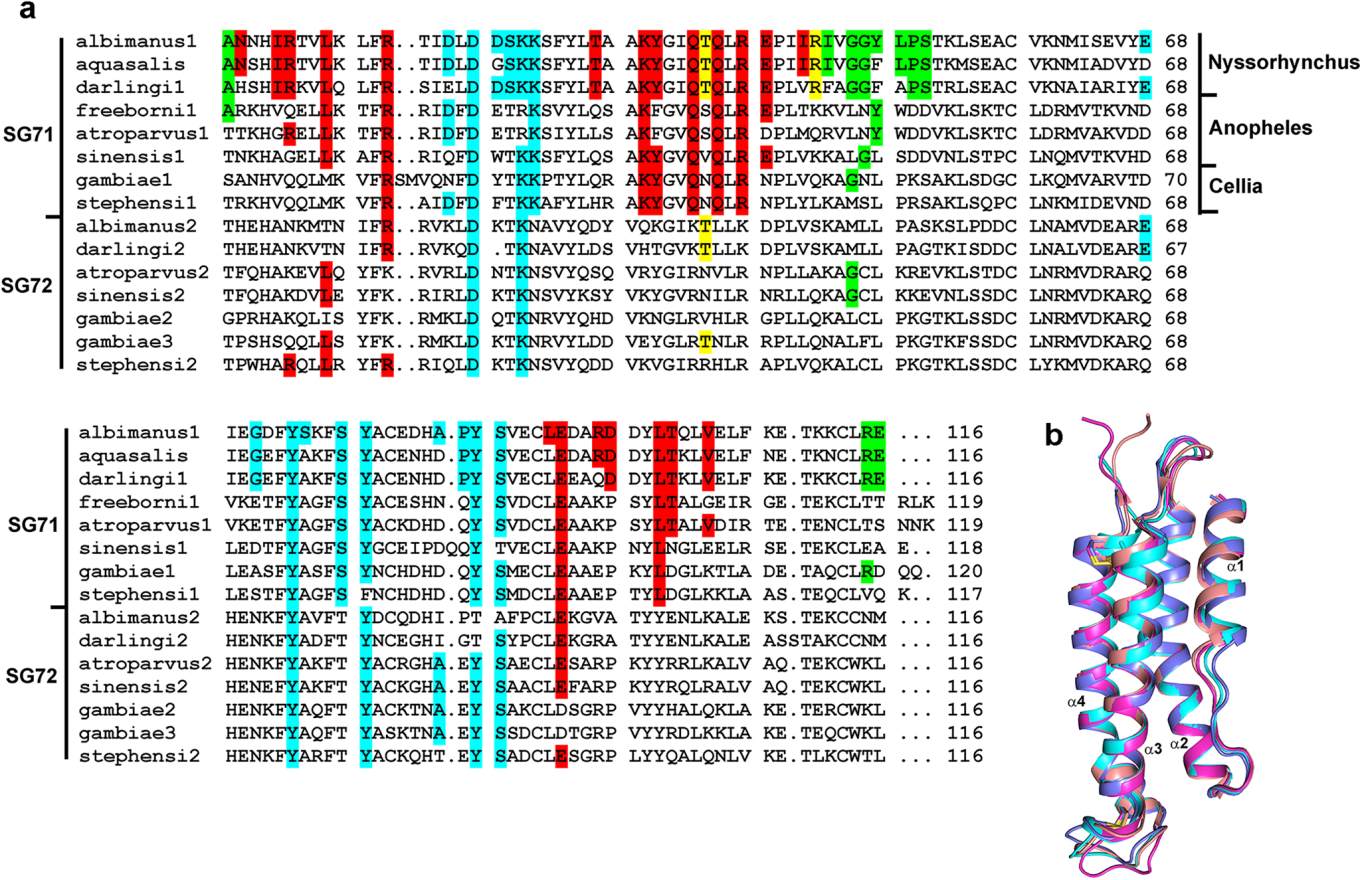
The Sg7 protein family from *Anopheles* mosquitoes. **a** Amino acid alignment of Sg7-1 and Sg7-2 proteins from the *Nyssorhynchus*, *Anopheles* and *Cellia* subgenera of the genus *Anopheles* showing residues involved in C3bBb binding as identified for the albicin complex using PISA. Identities in the binding interface are shaded. Residues interacting with the C3b α-chain or both the α-chain and β-chain are shaded in red, those interacting with the β-chain alone are shaded in yellow, those interacting with the α-chain of the second monomer are shaded in green, and those interacting with FBb are shaded in cyan. **b** Superposition of Sg7-1 models from species representing the three major subgenera of *Anopheles*. The figure includes the crystal structures of albicin (PDB: 6XKE, light blue) from *An. albimanus* and SG7AF (PDB: 6XL7, copper) from *An. freeborni* (RMSD with albicin 0.71 Å over 96 Cα positions) along with models of Sg7-1 from *An. atroparvus* (magenta, RMSD with albicin 0.69 over 100 Cα positions) and *An. stephensi* (cyan, RMSD with albicin 0.84 Å over 109 Cα positions) generated with Alphafold 2. Disulfide bonds are shown as sticks with sulfur in yellow.

*Anopheles* species contain tandemly duplicated Sg7 genes on chromosome 3 which have been given the names Sg7-1 and Sg7-2^32^. A third gene is also present in the sibling species *An. gambiae* and *An. coluzzii*^32^. Albicin and other complement inhibitors belong to the Sg7-1 clade which shows sequence identities of 45-90% within the clade and approximately 35% with members of the Sg7-2 clade (Fig. 8a)^32^. Recombinant Sg7-2 from *An. albimanus* has been tested and found to be inactive in erythrocyte lysis and SPR assays^7^. Alignment of Sg-7 sequences from *Anopheles* species shows high degree of sequence identity in the C3bBb binding interface (as determined using PISA^29^) between albicin and other Sg7-1 forms in the subgenus *Nyssorhynchus* (Fig. 8a). The more distantly related *Anopheles* and *Cellia* subgenera contain fewer amino acid sequence identities with albicin in the binding interface (Fig. 8a). Previously determined crystal structures and structure predictions made using Alphafold 2 show almost identical backbone conformations for the Sg7-1 proteins albicin from *An. albimanus* (*Nyssorhynchus*), Sg7AF from *An. freeborni* (*Anopheles*), Sg7-1 from *An. atroparvus* (*Anopheles*) and anophensin from *An. stephensi* (*Cellia*) but amino acid side chain differences at multiple positions could be responsible for the observed differences in potency (Fig. 8a, b). For instance, in the region where albicin interacts with MG7 of C3b monomer 2, Arg 115 is conserved in *Nyssorhynchus* species but is replaced by threonine in *An. freeborni* and valine *An. stephensi* thereby removing a possible salt bridge interaction with Glu 837 of the MG7 domain indicated by our models. Additionally, Leu 49 at the apex of the α2-α3 loop of albicin is replaced by tryptophan in *An. freeborni* which, without significant rearrangement of adjacent residues, would appear to cause a steric clash between the inhibitor and C3b. In the region where albicin interacts with FBb, Asp 19, at the apex of the α1-α2 loop, forms potential electrostatic interactions with Gln 411 and Lys 429 in the vWA domain of FBb. This residue is substituted by phenylalanine in *An. stephensi* resulting in loss of these potential interactions. Notably, *An. freeborni* contains glutamate at position 19, suggesting possible importance of an acidic side chain at this position for inhibitory activity.

## Discussion

Using cryo EM we have determined the structures of the transient complexes of C3bBb inhibited by the mosquito salivary protein albicin. The mechanism of inhibition is much like that of the staphylococcal inhibitor SCIN but the binding interfaces differ in the two inhibitory systems. Convergent evolution has apparently led these two unrelated molecules to form interactions with C3b in the MG6-MG7 regions and FBb in the vWA domain which promote formation of a dimeric C3bBb complex containing bound inhibitor. Evolution of this type of mechanism is apparently assisted by the favorable symmetrical interaction of C3b monomers through their MG4 and MG5 domains. These interacting domains make up part of the binding interface for the substrate C3 with C3bBb but the interaction of C3b or C3bBb monomers is not sufficiently strong to result in accumulation of C3b or C3bBb dimers in the absence of inhibitors^22^. Albicin and SCIN further stabilize the interface by forming bridging contacts involving both monomers at the end of the C3b dimer opposite MG4 and MG5. This results in a C3b-C3b complex that is tethered by extensive binding interactions at each end of the dimer axis of symmetry.

We have shown that FBb is essential to the formation of an inhibited complex through high affinity binding of albicin^7,19^. The structures determined after a 30 min. incubation time are made up of albicin bound intermediates containing one or two molecules of FBb as well as a C3b dimer class containing no bound FBb. This suggests that FBb dissociates relatively quickly from the complex leaving a C3b-albicin dimer that is stable enough to accumulate in the mixture. The multiphasic kinetics observed for the dissociation of C3bBb-albicin in surface plasmon resonance experiments almost certainly reflect the decay of these multiple inhibited complex forms^7,19^

This requirement for the presence of FBb to allow for albicin binding likely goes beyond the provision of an additional binding surface for the inhibitor. Specific recognition of the C3b-bound, activated protease allows the inhibitor to distinguish between the mature proteolytic complex and free C3, C3b, iC3b and C3c which would be potential targets and may be considerably more abundant at the feeding site. This selectivity would prevent wastage of the available salivary protein on the formation of C3b dimers while maximizing the inactivation of C3bBb. It seems likely that the strength of the molecular interactions in these complexes are tuned to maximize interaction with preferred targets while reducing the likelihood of binding with non-preferred targets. These considerations are especially important for salivary inhibitors, which are present in small amounts and are injected into a relatively large volume of host blood. To be effective, the local inhibitor concentration must approximate that of its molecular target; in this case the C3bBb monomer.

The function of salivary complement inhibition in blood feeding has not been firmly established but the effects of activated complement can be deleterious in several ways. Anaphylatoxins C3a and C5a are highly potent proinflammatory factors which would activate immune cells, promote plasma leakage and cause pain leading to ingestion of a less nutritious meal and possibly eliciting host behavioral responses that would interrupt feeding^33^. Activated complement in the blood meal could also be damaging to midgut tissue through formation of the MAC. A significant proportion of injected saliva ingested along with the blood meal so a salivary inhibitor has the potential to act in the host and in the insect midgut and albicin could be functioning in either one of these environments to benefit blood feeding or digestion. Complement activation in the mosquito gut is also known to be important for parasite killing in some cases^34^. The efficacy of an important transmission-blocking vaccine candidate for *Plasmodium falciparum* malaria, Pfs320, has been found to be dependent on complement fixation, particularly in humans and other primates^11,34^. The presence of re ingested salivary complement inhibitors in the midgut of inhibitor producing mosquito species could complicate the development of vaccines in these cases.

The observed interspecies variability in complement inhibition is curious since all *Anopheles* species contain the Sg7-1 gene and appear to express the protein in salivary glands^32^. Based on assay of salivary gland extracts, species in the subgenus *Cellia* contain little or no anticomplement activity^7^. Conversely, extracts from species in the subgenera *Nyssorhynchus* and *Anopheles* are inhibitory^7,19^. Experiments with gland extracts have been backed up in several instances by experiments with recombinant Sg7-1 proteins from *An. albimanus*, and *An. darlingi* of the *Nyssorhynchus* subgenus as well as *An. freeborni* of the *Anopheles* subgenus showing potent activity. The orthologous protein from *An. stephensi* of the *Cellia* subgenus shows dramatically reduced activity. Phylogenetic analyses of *Anopheles* species indicate that *Nyssorhynchus* split from the *Cellia/Anopheles* lineage approximately 100 million years ago, suggesting that anticomplement function of Sg7-1 proteins was present in the common ancestor of the three subgenera^35^. Apparently, in *Cellia* species this function has been lost or diminished, perhaps because of the evolution of alternative mechanisms of complement inhibition or host preference considerations. One member of this subgenus, *An. gambiae*, has been shown to bind the complement control protein factor H from the host blood meal on receptors in the midgut^36^. It is not presently known how widely this mechanism is distributed throughout the genus. Additionally, anopheline mosquitoes show host selectivity differences that could impact salivary function in response to structural differences in host C3b and FB^37^.

## Methods

### Proteins

Albicin was produced as inclusion bodies in *Escherichia coli*, refolded and purified as described previously^7,19^. Human C3b (catalog number A114), FB (A135) and FD (A136) were obtained from Complement Technology.

### Complex formation, grid preparation and image collection

C3bBb-albicin complexes were formed by mixing C3b (570 nM), FB (570 nM), FD (150 nM) and albicin (2.8 µM) in 10 mM HEPES pH 7.4, 150 mM NaCl, 5 mM MgCl_2_ (buffer A) and incubating at room temperature 30 min. The C3bB complex was formed by mixing C3b, FB and albicin in buffer A with MgCl_2_ replaced by NiCl_2_ or buffer A-Ni. Size exclusion chromatography was performed on a Superdex 200 increase 10/300 column (Cytiva) equilibrated with buffer A or buffer A-Ni. SDS PAGE was performed in 10% BisTris gels (Thermo) which were stained with silver. SPR measurements were perfomed on a Biacore T200 instrument (Cytiva) with a C3b surface immobilized to a level of 1000 RU at pH 5.0 on a CM5 chip (Cytiva) by amine coupling. The experiments were performed in buffer A at albicin concentrations between 0.094 and 12 µM. For cryo EM grid preparation, complexes were concentrated by ultrafiltration through Amicon centrifugal filters (10 kDa MW cutoff) to a concentration of approximately 1.5 mg/mL and 3 µL of protein sample was applied to freshly glow-discharged (easiGLow) C-flat grids (Protochips, CF1.2/1.3-3Au). Blotting was performed at 6 °C and 100% humidity using a Vitrobot Mark IV (Thermo-Fisher), with a 2.5 s blotting time and 4 pN blotting force. Grids were then plunged into liquid ethane and stored in liquid nitrogen before examination by cryo-EM. Images were recorded on a Glacios TEM (Thermo Fisher) at 200 kV and recorded at 45,000X, on a K3 direct electron detector (Gatan) in super-resolution mode.

### Cryo-EM Image processing

Movies were processed with MotionCor2 (Table 1)^38^, during which dose weighting was applied and the pixel size was binned to 0.88 Å/pixel. The contrast transfer function (CTF) was estimated in Ctffind4^39,40^. Particle picking was conducted in Gautomatch (http://www.mrc-lmb.cam.ac.uk/kzhang/Gautomatch/) using references generated with EMAN2^41^ resulting in a set of 492,352 particles (Table 1, Supplementary Fig. S2). These were extracted in RELION-4.0.1^42^ with a box size of 320 ×320 pixels. The picked-particles were subjected to 2D classification in RELION-4.0.1 to remove bad particles (Supplementary Fig. S2). An initial reference structure was used as a template for 3D classification. Three classes were selected for subsequent gold-standard refinement (FSC = 0.143) in RELION-4.0.1 followed by CTF refinement and particle polishing, also in RELION (Table 1, Supplementary Fig. S2). Further refinement using uniform and nonuniform protocols in cryoSPARC^39^ with C1 or C2 symmetry applied resulted in final resolutions for reconstructions containing zero (class 4), one (class 1) or two (class 3) molecules of FBb 3.59 Å, 4.42 Å and 3.86 Å, respectively (Table 1, Supplementary Fig. S2). Particle subtraction and localized refinement routines in cryoSPARC were used to improve maps at the albicin-C3bBb interfaces.

#### Model building and refinement of C3bB and C3bB-lufaxin complexes

Models of C3bBb and C3b in the dimeric arrangement were obtained from the crystal structure of the C3bBb-SCIN complex (PDB accession code 6RUR^21^) and the crystal structure albicin (PDB accession code 6XKE^19^). Positioning was assisted by the Phenix dock-in-map application or ChimeraX^43,44^. Models were refined using real-space refinement in Phenix combined with manual rebuilding using Coot^43,45^ (Table 1). Model quality was evaluated using MolProbity^46^ (Table 1). Figures were generated with UCSF ChimeraX^44^ and Pymol (Schrodinger Inc). Identification of interfaces and buried surface regions in the various complexes was done using PISA^29^. Alphafold2 (2.3.2^47^) was run on the Biowulf HPC cluster at the National Institutes of Health using input amino acid sequences derived from translation of publicly available genomic data (predicted open reading frames) or cDNA transcripts. The predicted signal sequences were deleted prior to structure prediction.

### Statistics and reproducibility

Surface plasmon resonance experiments measuring the concentration dependence of albicin binding with immobilized C3b were performed as of a single concentration series with single samples of the proteins and analyzed using the non-linear regression package in the Biacore T200 Evaluation software (version 3.2, Cytiva).

### Reporting summary

Further information on research design is available in the Nature Portfolio Reporting Summary linked to this article.

### Supplementary information

Supplementary information

Description of Additional Supplementary Materials

Supplementary Data 1

Supplementary Data 2

Reporting Summary

## Data Availability

The cryo-EM 3D maps for the C3b-albicin and C3bBb-albicin structures were deposited in EMDB database with the accession codes EMD-42244 and EMD-42300, respectively. The corresponding atomic coordinates were deposited in the PDB with the accession codes 8UH2 and 8UIN, respectively. The raw images for all SDS-PAGE gels contained in Fig. 1 and Supplementary Fig. S1 are shown in Supplementary Fig. S10. Supplementary Data 1 contains raw detector output data (280 nm) for chromatography runs in Fig. 1 and Supplementary Fig. S1. Supplementary Data 2 contains raw surface plasmon resonance numerical output for Supplementary Figs. S1d and S1e, output for non-linear fitting to a hyperbolic model for equilibrium binding, and fitting statistics and parameters (equilibrium binding constant) from the non-linear regression There are no restrictions on data availability.
