# Supplementary material for: Mechanism of complement inhibition by a mosquito protein revealed through cryo-EM
Source: Commun Biol. 2024 May 27;7:649. doi: 10.1038/s42003-024-06351-x (PMC11130238; doi:10.1038/s42003-024-06351-x)
Supplement: Supplementary file 1 — Supplementary information [file 42003_2024_6351_MOESM1_ESM.pdf]

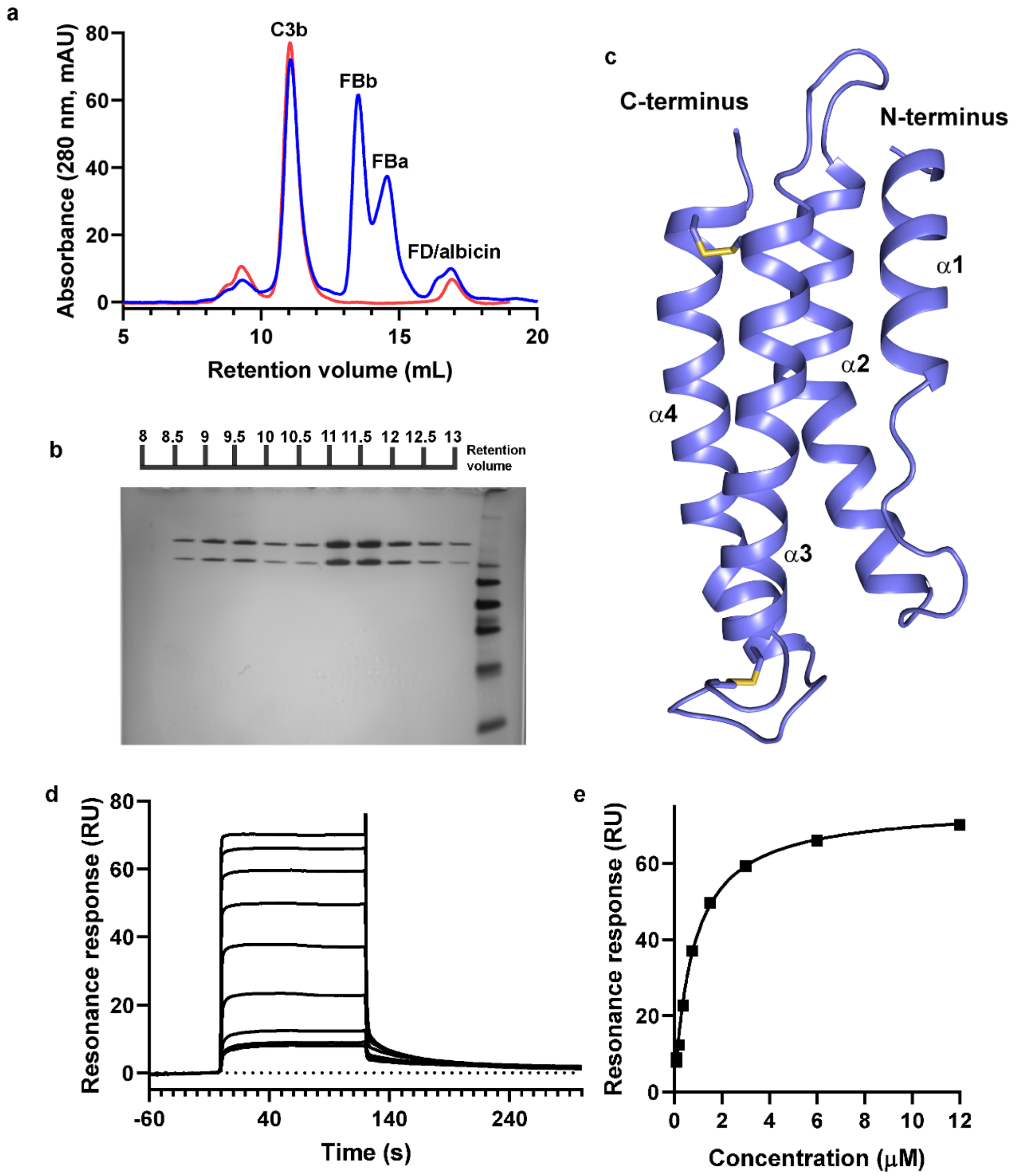


Fig. S1. Size exclusion chromatography of a C3b-albicin mixture. a) Chromatogram is a reproduction of Fig. 1d showing the retention volumes of C3b and albicin. b) SDS-PAGE of 0.5 mL fractions shows that no albicin is associated with eluted C3b. c) Structure of albicin showing the helical elements labeled and cysteine residues participating in disulfide bonds as sticks (with sulfur atoms colored yellow). d) SPR traces showing albicin binding with a C3b surface. Concentrations of albicin analyte are 0.094, 0.187, 0.375, 0.75, 1.5, 3.0, 6.0 and 12.0 μM. e) Fitting of equilibrium resonance values to a binding function yields a $K_D$ value of 0.8 μM

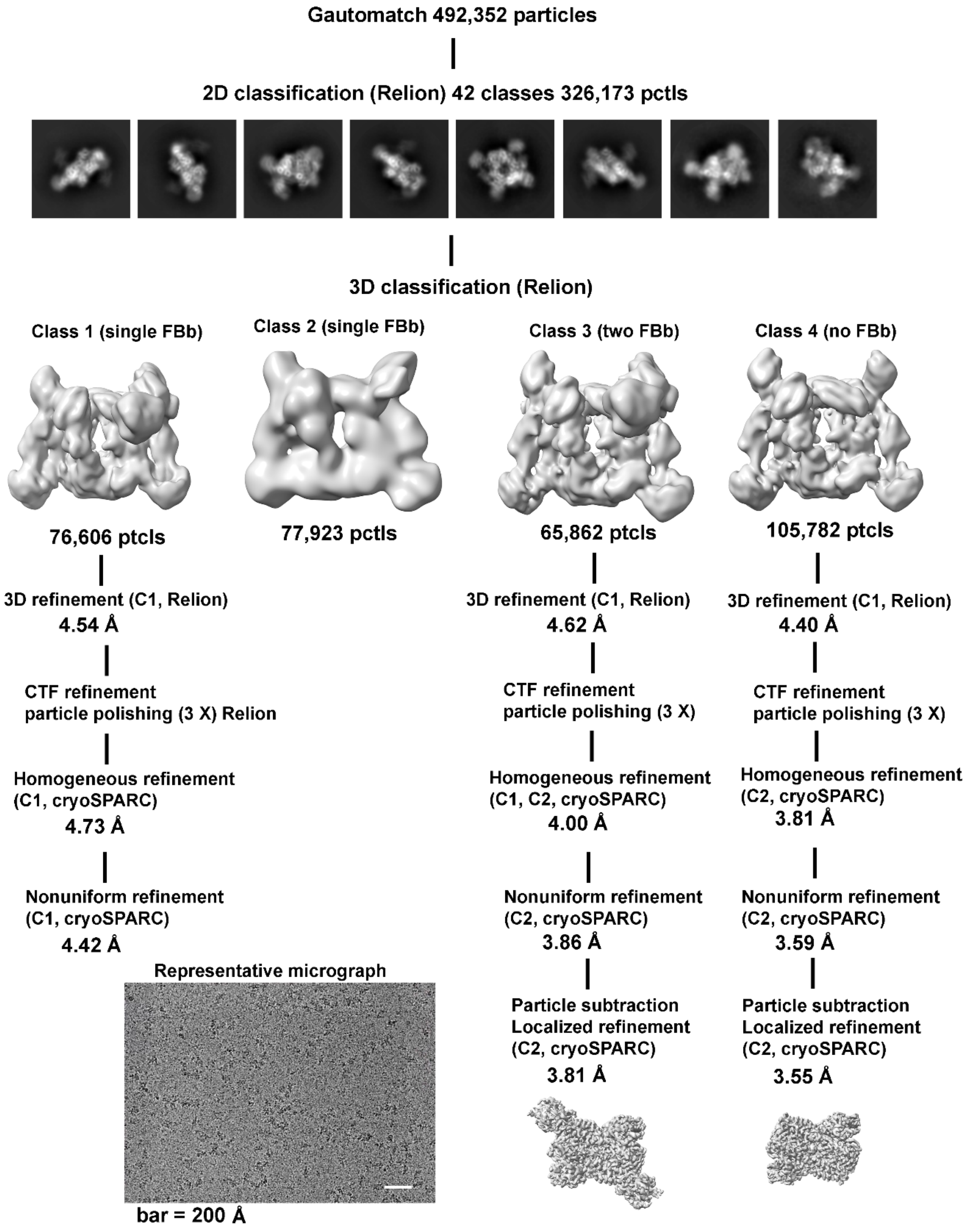


Fig. S2 Workflow for cryo EM data processing, including a representative micrograph (lower left).

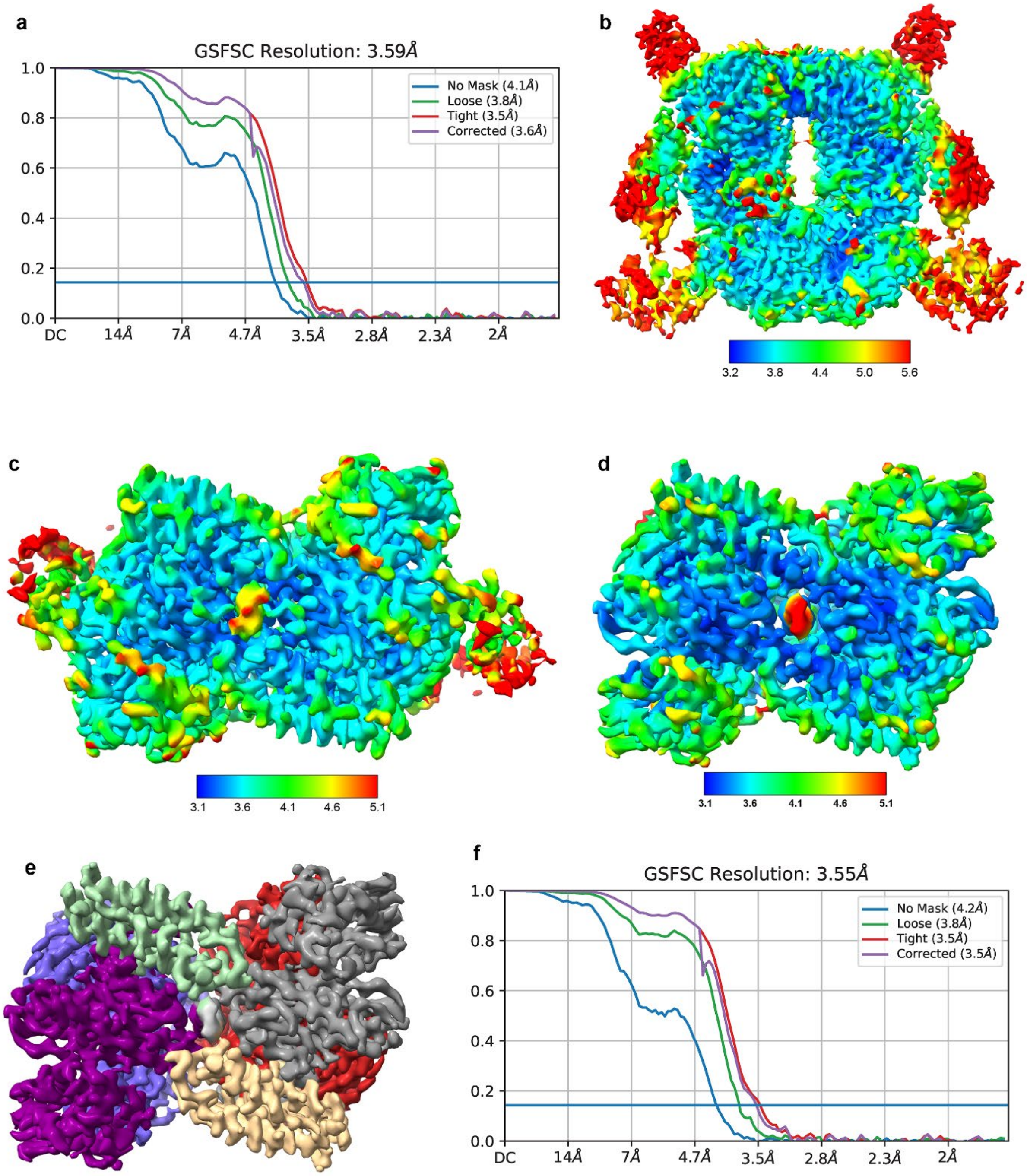


Fig. S3 Refinement of C3b-albicin dimeric structure. a) FSC curve after non uniform refinement. b) Local resolution map calculated in cryoSPARC after non uniform refinement. c) Local resolution map of the albicin interaction interfaces after non uniform refinement. d) Local resolution map of the albicin interaction interfaces after localized refinement. e) Map after localized refinement with regions colored by the associated protein chains: albicin, green/wheat; C3b β-chain, blue/red; C3b α-chain purple/grey. f) FSC curve after localized refinement.

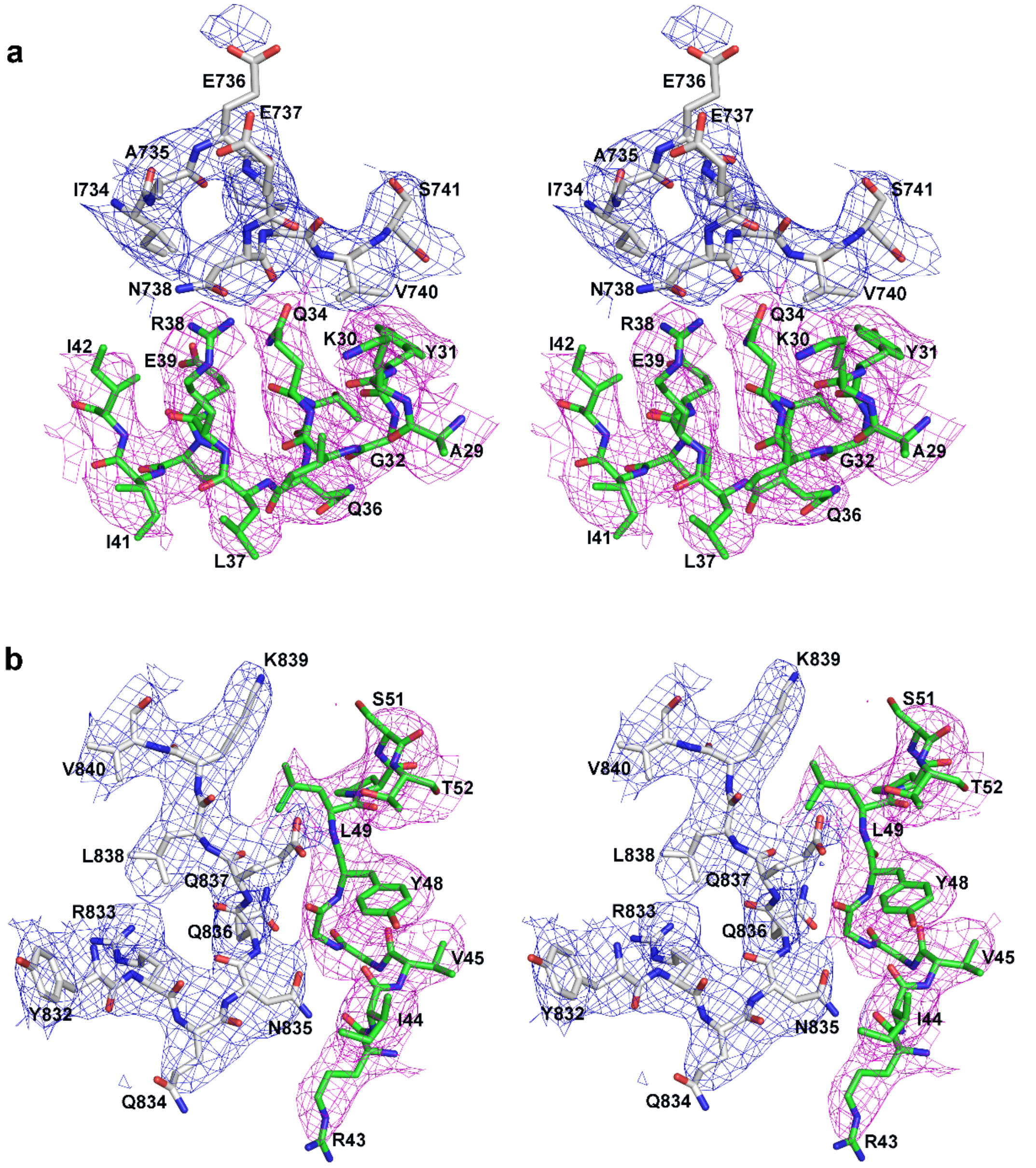


Fig. S4. Representative density covering interface residues. a) Stereoview of density covering in albicin (green, sticks, magenta, density) and C3b (grey, sticks, blue, density) in the interface region of monomer A. b) Stereoview of density covering in albicin (green, sticks, magenta, density) and C3b (grey, sticks, blue, density) in the interface region of monomer B.

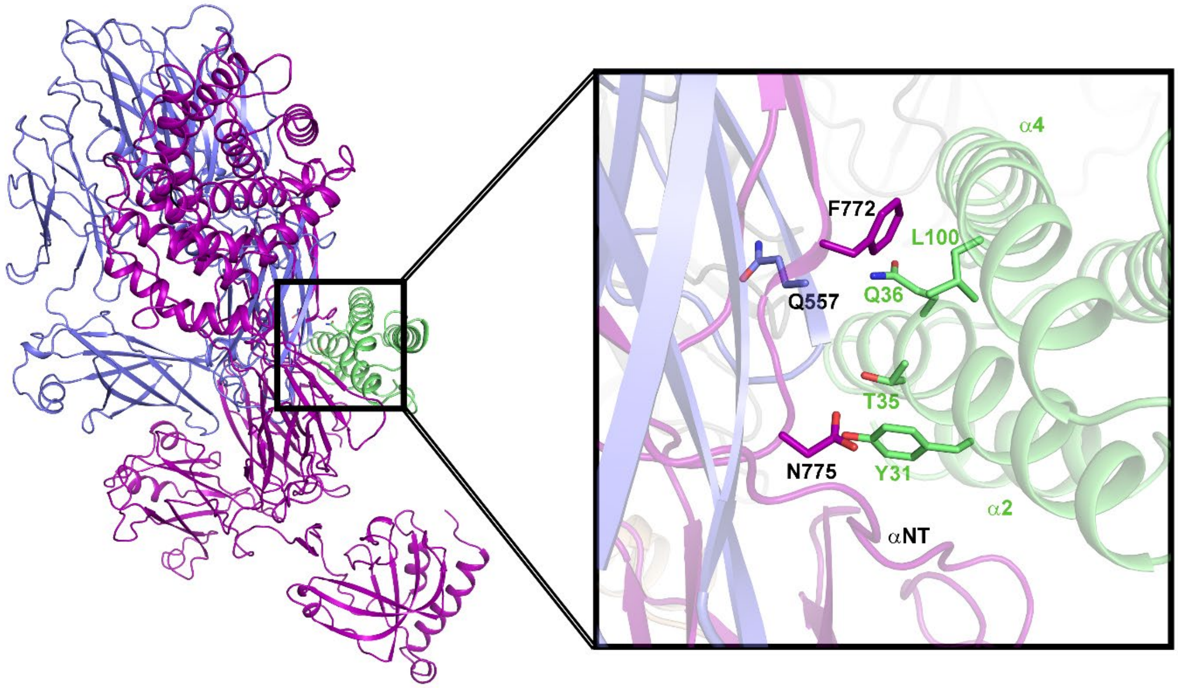


Fig. S5. Detail of interactions of the α2-α4 face of albicin (green) with the MG6 domain (α-chain, purple, β-chain, blue) of C3b.

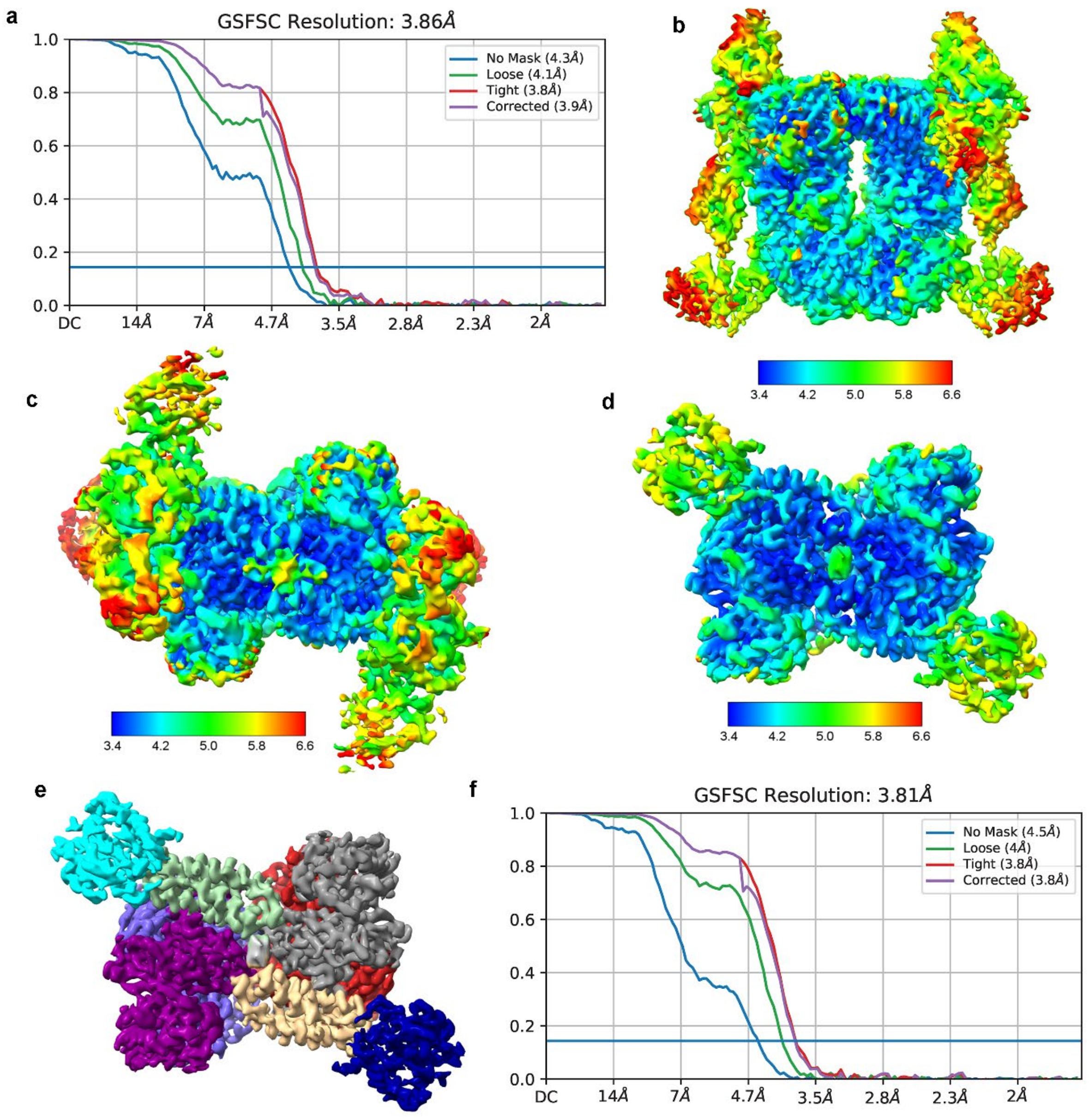


Fig. S6. Refinement of C3bBb-albicin dimeric structure. a) FSC curve after non uniform refinement. b) Local resolution map calculated in cryoSPARC after non uniform refinement. c) Local resolution map of the albicin interaction interfaces after non uniform refinement. d) Local resolution map of the albicin interaction interfaces after localized refinement. e) Map after localized refinement colored according to the associated protein chains: albicin, green/wheat; C3b β-chain, blue/red; C3b α-chain, purple/grey; FBb (vWA domain), dark blue and cyan. f) FSC curve after localized refinement.

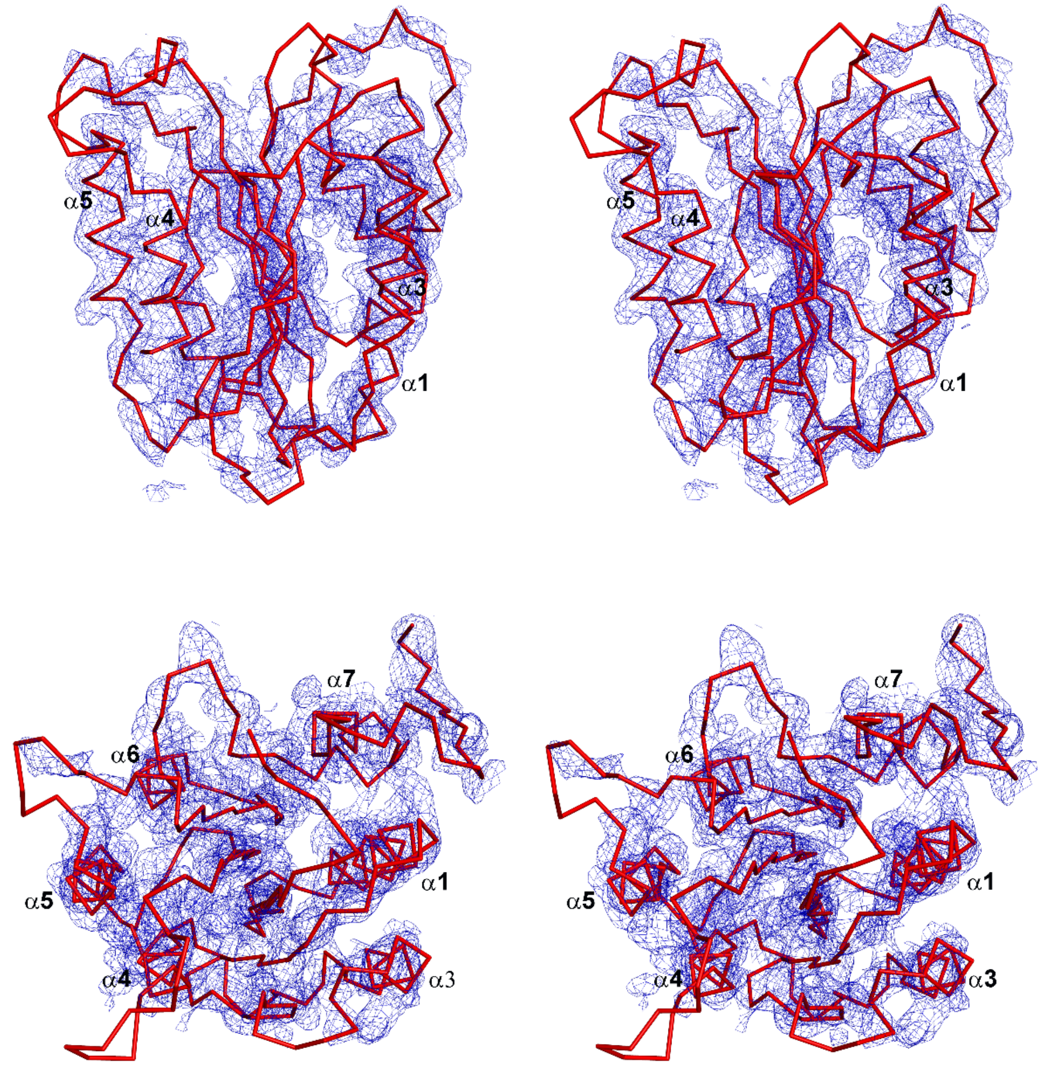


Fig. S7. Stereoviews of density (after localized refinement) covering the vWA domain of FBb (red Cα trace). α-helical elements are labeled.

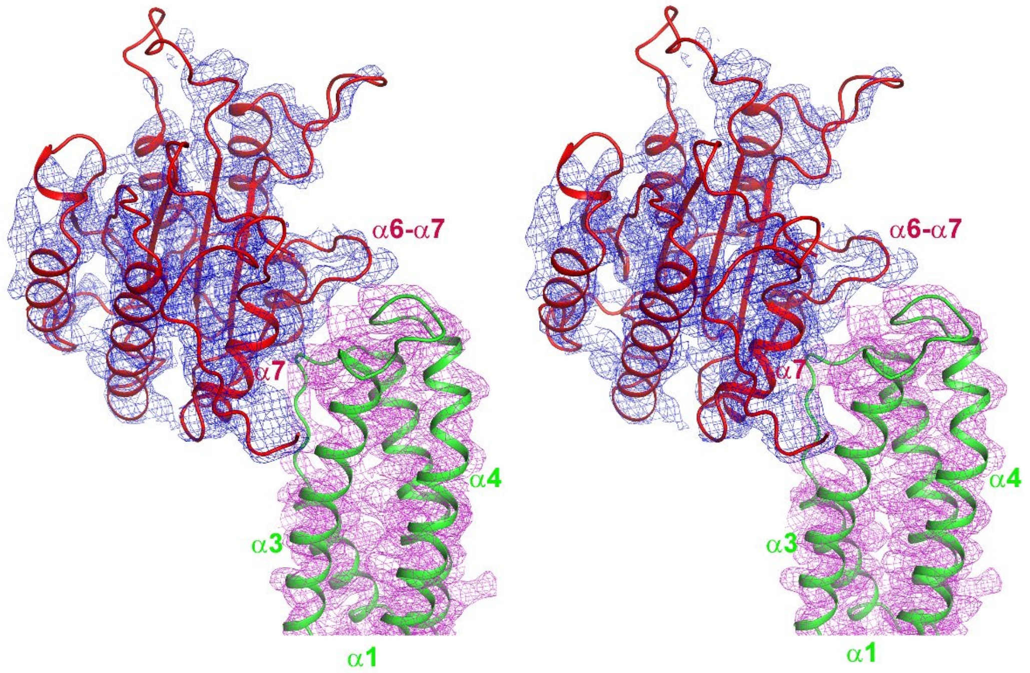


Fig. S8. Stereoview of density (after localized refinement) covering the lufaxin (green ribbon) and FBb vWA domain (red ribbon) interface. α-helical and loop elements are labeled.

**a**

```
   scin   .......... ...STSLPTS N.......E Y.QNEKLANE LKSLLD.ELN
albicin   ANNHIRTVLK LFRTIDLDDS KKSFYLTAAK YGIQTQLREP IIRIVGGYLP

   scin   VNELATGSLN TYYKRTIKIS G.LKAMYALK SKD.....FK KMSEAK..Y.
albicin   STKLSEACVK NMISEVYEIE GDFYSKFSYA CEDHAPYSVE CLEDARDDYL

   scin   .QLQKIYNEI DEALKSKY
albicin   TQLVELFKET KKCLRE..
```

**b** **c**

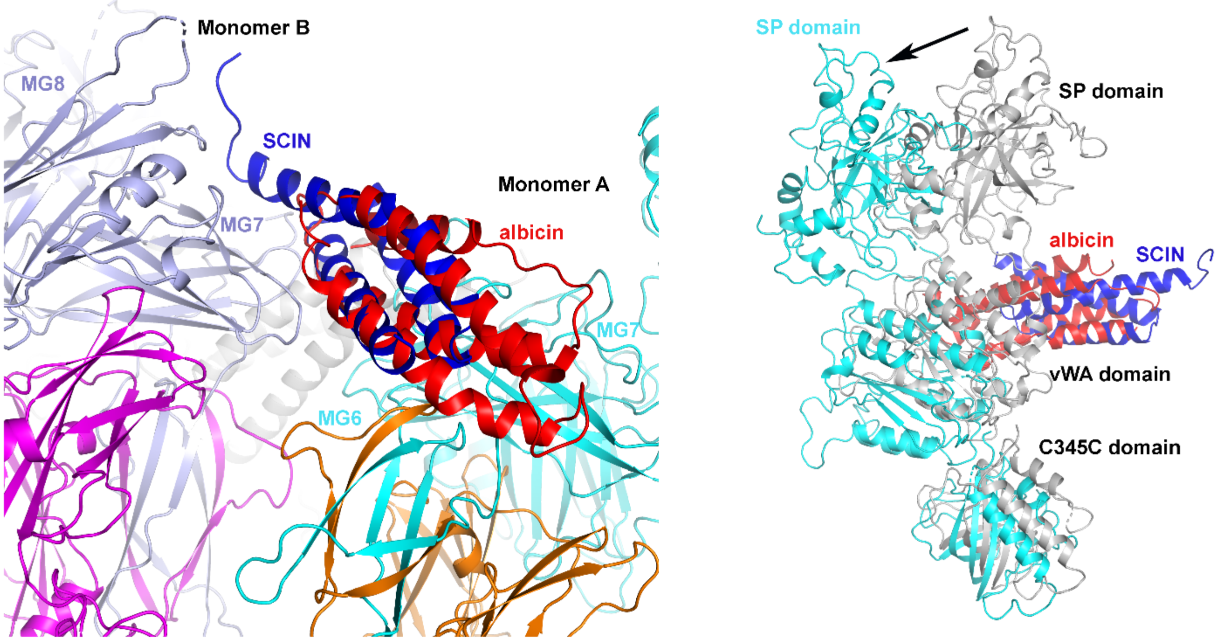


Fig. S9. Sequence alignment and comparison of albicin and SCIN binding modes with C3bBb. a) Pairwise Clustal alignment of the amino acid sequences of SCIN and albicin. The alignment shows amino acid identity of 18% and results in insertion of several gaps. b), Albicin (red) from monomer A extends further along the C3b surface than SCIN (blue) covering more of MG6 and MG7 of monomer A. At the same time SCIN extends further in the opposite direction and forms more extensive contacts with MG7 of monomer B. c) The positioning of albicin pushes the C345C, vWA and SP domains of FBb (cyan) away from the C3b surface, relative to the SCIN complex (grey).

**Fig. 1b**

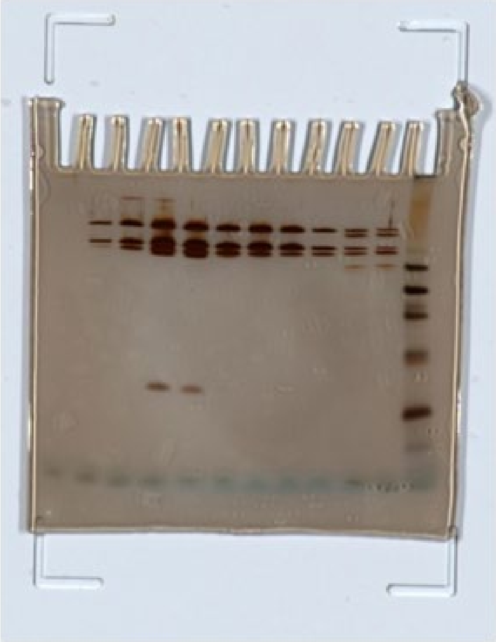

**Fig. 1c**

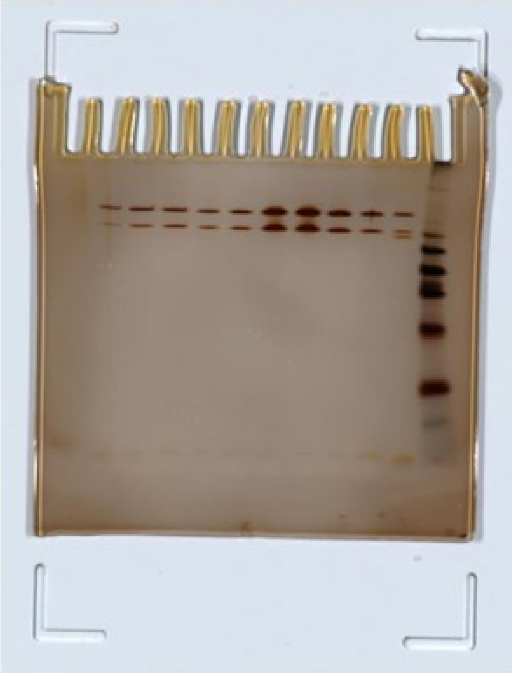

**Fig. 1f**

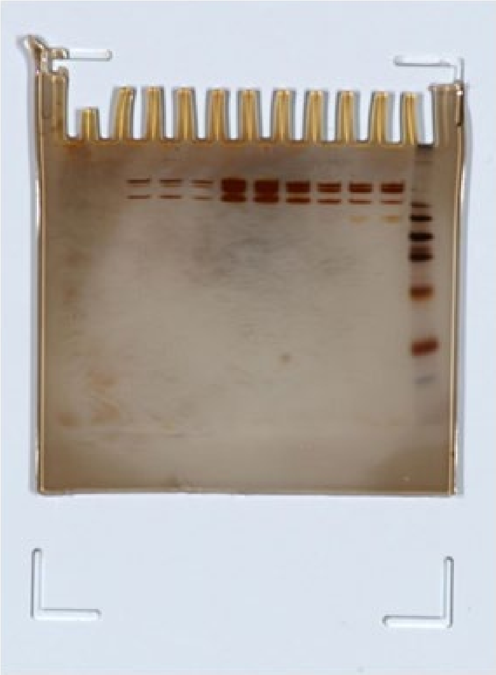

**Fig. S1b**

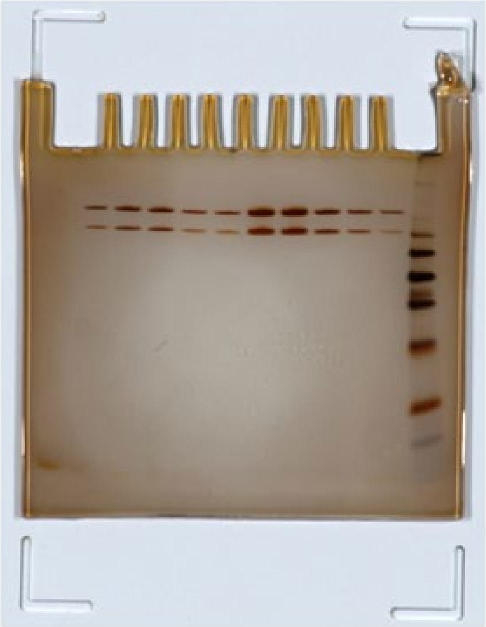

Fig. S10. Raw images of SDS PAGE gels for Fig. 1b, Fig. 1c, Fig. 1f and Fig. S1b.

Table. S1. Interfacial residues for albicin with C3b and FB showing chain, residue number and buried surface area (BSA, Å$^2$) as calculated using PISA.

A. α-chain C3b (chain H monomer A) with albicin (chain C)

| Chain--Residue--BSA | Chain--Residue--BSA |
|---|---|
| H:GLU 731 4.31 | C:ASN 2 42.83 |
| H:ASP 732 48.91 | C:ILE 5 22.26 |
| H:ILE 733 1.84 | C:ARG 6 36.46 |
| H:ILE 734 25.93 | C:LEU 9 39.16 |
| H:ALA 735 12.70 | C:ARG 13 60.09 |
| H:GLU 737 10.80 | C:THR 27 30.87 |
| H:ASN 738 69.15 | C:LYS 30 21.60 |
| H:ILE 739 5.86 | C:TYR 31 97.38 |
| H:VAL 740 103.26 | C:GLN 34 47.81 |
| H:SER 741 5.48 | C:GLN 36 13.62 |
| H:ARG 742 16.40 | C:ARG 38 69.22 |
| H:SER 743 1.73 | C:GLU 39 15.87 |
| H:GLU 744 31.93 | C:ILE 42 22.59 |
| H:ASN 770 26.94 | C:LEU 92 0.50 |
| H:ILE 771 0.67 | C:GLU 93 28.33 |
| H:PHE 772 131.11 | C:ARG 96 24.38 |
| H:LYS 774 21.77 | C:ASP 97 36.49 |
| H:ASP 775 34.00 | C:LEU 100 31.84 |
| H:HIS 896 12.25 | C:THR 101 21.80 |
| H:PHE 898 106.12 | C:VAL 104 8.55 |
| H:ILE 899 11.88 | |
| H:SER 900 16.21 | |
| H:ARG 904 21.12 | |

B. β-chain C3b (chain G monomer A) with albicin

| Chain--Residue--BSA | Chain--Residue--BSA |
|---|---|
| G:ARG 551 26.05 | C:TYR 31 26.04 |
| G:VAL 554 20.64 | C:THR 35 26.61 |
| G:PRO 555 22.38 | C:GLN 36 32.96 |
| G:GLY 556 54.58 | C:GLU 39 4.90 |
| G:GLN 557 5.82 | C:ARG 43 44.24 |
| G:GLN 558 41.42 | C:LEU 100 6.36 |
| | C:THR 101 6.81 |
| | C:VAL 104 22.16 |

C. α-chain C3b (chain B monomer B) with albicin

| Chain--Residue--BSA | Chain--Residue--BSA |
|---|---|
| B:GLN 834 0.37 | C:ALA 1 4.18 |
| B:ASN 835 88.86 | C:ARG 43 34.26 |
| B:GLN 836 23.81 | C:ILE 44 20.57 |
| B:GLU 837 93.02 | C:GLY 46 5.69 |
| B:LEU 838 1.55 | C:GLY 47 63.96 |
| B:LYS 839 21.65 | C:TYR 48 6.43 |
| B:THR 865 14.23 | C:LEU 49 90.35 |
| B:PRO 867 8.54 | C:PRO 50 7.36 |
| B:PRO 868 10.38 | C:SER 51 4.66 |
| B:HIS 896 7.80 | C:ARG 115 45.16 |
| B:PHE1416 26.89 | C:GLU 116 32.88 |

D. FBb (chain J monomer A) with albicin

| Chain--Residue--BSA | Chain--Residue--BSA |
|---|---|
| J:VAL 409 0.17 | C:ASP 16 21.09 |
| J:GLN 411 47.69 | C:ASP 18 40.29 |
| J:ASN 415 1.45 | C:ASP 19 48.26 |
| J:LYS 420 12.05 | C:SER 20 94.29 |
| J:LYS 421 0.24 | C:LYS 21 2.51 |
| J:ASN 423 80.63 | C:LYS 22 53.97 |
| J:GLU 424 21.50 | C:GLU 68 3.93 |
| J:GLN 425 67.75 | C:GLY 71 0.74 |
| J:VAL 427 21.79 | C:TYR 74 23.00 |
| J:PHE 428 12.65 | C:SER 75 28.54 |
| J:LYS 429 54.08 | C:SER 78 1.20 |
| J:LYS 431 23.24 | C:TYR 79 111.07 |
| J:ASP 432 6.27 | C:ALA 85 8.37 |
| J:ASP 438 9.47 | C:PRO 86 25.14 |
| J:TYR 441 37.15 | C:TYR 87 92.43 |
| J:GLN 442 70.44 | C:SER 88 3.51 |
| J:HIS 459 13.29 | |
| J:ARG 460 3.78 | |
| J:LYS 461 46.69 | |

E. α-chain C3b (chain H monomer A) with SCIN (chain Q)

| Chain--Residue--BSA | Chain--Residue--BSA |
|---|---|
| H:ASP 730 54.45 | Q:GLU 30 0.86 |
| H:GLU 731 8.78 | Q:ASN 37 34.53 |
| H:ASP 732 44.79 | Q:THR 38 33.02 |
| H:ILE 733 5.40 | Q:TYR 40 11.59 |
| H:ILE 734 26.28 | Q:LYS 41 14.22 |
| H:ALA 735 10.22 | Q:ARG 42 98.73 |
| H:GLU 737 14.11 | Q:LYS 45 63.32 |
| H:ASN 738 80.55 | Q:ILE 46 90.25 |
| H:ILE 739 0.67 | Q:GLN 49 98.52 |
| H:VAL 740 97.35 | Q:LYS 50 43.49 |
| H:SER 741 9.57 | Q:TYR 53 86.59 |
| H:ARG 742 20.92 | Q:LYS 56 27.99 |
| H:GLU 744 15.82 | Q:LYS 62 17.04 |
| H:PHE 772 44.37 | Q:GLN 69 5.16 |
| H:ASP 775 20.58 | |
| H:HIS 896 3.13 | |
| H:PHE 898 109.90 | |
| H:ILE 899 3.52 | |
| H:SER 900 21.41 | |
| H:ASP 901 0.49 | |

F. β-chain C3b (chain G monomer A) with SCIN

| Chain--Residue--BSA | Chain--Residue--BSA |
|---|---|
| G:VAL 554 7.86 | Q:ASN 37 2.01 |
| G:PRO 555 25.42 | Q:TYR 39 69.27 |
| G:GLY 556 37.16 | Q:TYR 40 17.20 |
| G:GLN 557 22.52 | Q:ARG 42 28.11 |
| G:GLN 558 17.80 | Q:ALA 80 1.00 |

G. α-chain C3b (chain B monomer B) with SCIN

| Chain--Residue--BSA | Chain--Residue--BSA |
|---|---|
| B:GLU 731 4.17 | Q:THR 2 3.43 |
| B:ASN 835 62.35 | Q:SER 3 15.35 |
| B:GLN 836 64.35 | Q:LEU 4 108.77 |
| B:GLU 837 95.20 | Q:PRO 5 24.86 |
| B:LEU 838 1.50 | Q:THR 6 29.21 |
| B:LYS 839 96.49 | Q:SER 7 63.65 |
| B:GLN 862 0.17 | Q:ASN 8 61.00 |
| B:THR 863 44.33 | Q:TYR 10 78.80 |
| B:VAL 864 4.32 | Q:GLN 11 70.53 |
| B:THR 865 35.40 | Q:ASN 12 4.08 |
| B:PRO 867 1.51 | Q:LYS 14 50.96 |
| B:PRO 868 26.92 | Q:LEU 15 5.70 |
| B:TYR 895 12.04 | Q:GLU 18 3.32 |
| B:HIS 896 75.90 | Q:ASP 59 21.25 |
| B:HIS 897 16.45 | Q:PHE 60 92.83 |
| B:ASP1357 3.43 | Q:LYS 61 81.89 |
| B:LYS1359 24.47 | Q:SER 64 29.17 |
| B:ASN1360 0.63 | Q:GLU 65 47.83 |
| B:ALA1415 15.73 | Q:LYS 67 9.33 |
| B:PHE1416 12.00 | Q:TYR 68 76.81 |
| B:SER1417 43.72 | Q:GLN 69 4.62 |
| B:ASP1418 0.50 | |
| B:GLN1443 12.49 | |
| B:TYR1444 58.02 | |
| B:PHE1445 49.48 | |
| B:ASN1446 84.24 | |
| B:VAL1447 2.15 | |

H. FBb (chain J monomer A) with SCIN

| Chain--Residue--BSA | Chain--Residue--BSA |
|---|---|
| J:LYS 235 11.18 | Q:ASP 24 11.30 |
| J:ASN 423 73.56 | Q:GLU 25 33.54 |
| J:GLU 424 8.68 | Q:ASN 27 93.58 |
| J:GLN 425 50.96 | Q:VAL 28 91.32 |
| J:PHE 428 3.28 | Q:ASN 29 19.54 |
| J:LYS 429 43.89 | Q:GLU 30 27.05 |
| J:LYS 431 20.67 | Q:LEU 31 120.75 |
| J:ASP 432 27.28 | Q:ALA 32 26.37 |
| J:GLU 434 0.12 | Q:GLY 34 60.02 |
| J:ASN 435 40.68 | Q:SER 35 63.58 |
| J:ASP 438 29.08 | Q:LEU 36 10.79 |
| J:VAL 439 7.30 | Q:ASN 37 21.10 |
| J:TYR 441 18.76 | Q:TYR 74 1.84. |
| J:GLN 442 78.63 | Q:ILE 77 0.50 |
| J:ARG 460 57.43 | Q:ASP 78 22.96 |
| J:LYS 461 130.39 | Q:LEU 81 23.46 |
| J:THR 463 10.87 | Q:LYS 82 6.86 |
| J:HIS 466 14.64 | Q:TYR 85 148.05 |
| J:LYS 562 17.36 | |
| J:TYR 563 1.01 | |
| J:GLY 564 9.92 | |
| J:GLN 565 19.68 | |
| J:THR 566 26.73 | |
| J:ILE 567 2.01 | |
