# Supplementary material for: Mechanism of complement inhibition by a mosquito protein revealed through cryo-EM
Source: Commun Biol. 2024 May 27;7:649. doi: 10.1038/s42003-024-06351-x (PMC11130238; doi:10.1038/s42003-024-06351-x)
Supplement: Supplementary file 2 — Description of Additional Supplementary Materials [file 42003_2024_6351_MOESM2_ESM.docx]

**Description of Additional Supplementary Files**

**File name:** Supplementary Data 1

**Description:** This is a tabbed excel spreadsheet containing time vs. absorbance data for the four chromatograms contained in figure 1 and figure S1.

**File name:** Supplementary Data 2

**Description:** This is a tabbed excel file containing raw data for SPR traces. Data for the plot of concentration vs. equilibrium RU value for each concentration as well as values for the fit line. Also included is a table of fitting statistics including estimation of the Kd parameter
